# Complement activation and M2-like macrophage accumulation in anti-MDA5 monoclonal antibody–induced hepatic injury in mice

**DOI:** 10.3389/fimmu.2026.1707202

**Published:** 2026-02-02

**Authors:** Takuma Koga, Yoshiaki Zaizen, Hiroyuki Suzuki, Suzuna Sugi, Hironao Hozumi, Noriho Sakamoto, Takafumi Suda, Hiroshi Mukae, Hironori Kusano, Akihiko Kawahara, Jun Akiba, Takumi Kawaguchi, Shinjiro Kaieda, Tomoaki Hoshino

**Affiliations:** 1 Division of Respirology, Neurology, and Rheumatology, Department of Medicine, Kurume University School of Medicine, Kurume, Japan; 2 Division of Gastroenterology, Department of Medicine, Kurume University School of Medicine, Kurume, Japan; 3 Second Division, Department of Internal Medicine, Hamamatsu University School of Medicine, Hamamatsu, Japan; 4 Department of Respiratory Medicine, Nagasaki University Graduate School of Biomedical Sciences, Nagasaki, Japan; 5 Department of Diagnostic Pathology, Kurume University Hospital, Kurume, Japan; 6 Department of Pathology, Kurume University School of Medicine, Kurume, Japan; 7 Cancer Innovation Laboratory (CIL), Center for Cancer Research (CCR), National Cancer Institute (NCI)-Frederick, Frederick, MD, United States

**Keywords:** anti-MDA5 antibody, complement activation, dermatomyositis, hepatic injury, macrophages

## Abstract

**Background:**

Patients with anti–melanoma differentiation–associated gene 5 (MDA5) antibody–positive dermatomyositis (DM) frequently develop rapidly progressive interstitial lung disease and may also exhibit hepatic dysfunction, yet the mechanisms of hepatic injury remain poorly defined. We investigated the roles of M2−like macrophages and complement activation in hepatic injury associated with anti−MDA5 antibody–positive DM.

**Methods:**

Liver specimens from five autopsy cases of anti-MDA5 antibody–positive DM were examined for the presence of CD80-positive M1-like and CD206-positive M2-like macrophages. To establish a model of antibody-mediated hepatic injury, human MDA5 transgenic mice were treated with in-house anti-human MDA5 monoclonal antibodies. The contribution of complement was assessed by comparing hepatic pathology between wild-type and complement component C3-deficient MDA5 transgenic mice. Liver tissues were analyzed by immunohistochemistry and western blotting, and single-cell RNA sequencing libraries were generated from snap-frozen mouse liver samples.

**Results:**

Autopsy liver specimens demonstrated the presence of CD80-positive M1-like and CD206-positive M2-like macrophages. In human MDA5 transgenic mice, administration of anti−human MDA5 monoclonal antibodies induced hepatic injury accompanied by increased infiltration of CD206-positive M2-like macrophages. This hepatic injury was markedly attenuated in C3-deficient MDA5 transgenic mice, supporting an important role for complement activation in this model.

**Conclusion:**

Complement activation and the accumulation of M2-like macrophages are associated with anti-human MDA5 monoclonal antibody–induced hepatic injury in mice. These findings provide mechanistic insight into antibody–complement–macrophage interactions and suggest that modulation of complement pathways may represent a potential therapeutic approach to limit liver and systemic involvement in this disorder.

## Introduction

Melanoma differentiation–associated gene 5 (MDA5) is a member of the RIG-I–like receptor family and functions as a cytosolic sensor of viral double-stranded RNA. Upon recognition of viral RNA, MDA5 activates downstream signaling through mitochondrial antiviral signaling protein (MAVS), leading to type I interferon production. Dysregulated MDA5 signaling and genetic variants have been implicated in several autoimmune diseases characterized by excessive type I interferon responses, including Aicardi–Goutières syndrome, systemic lupus erythematosus, and type 1 diabetes mellitus (1–5).

Dermatomyositis (DM) is an autoimmune disease affecting skeletal muscle and skin and is frequently complicated by interstitial lung disease (ILD). A subset of patients presents with characteristic cutaneous findings but minimal muscle involvement, a condition known as clinically amyopathic DM (CADM). ILD, particularly rapidly progressive ILD (RP-ILD), is a major determinant of prognosis in DM and is especially common and severe in CADM (6). Recent studies have identified autoantibody-defined subgroups of DM, among which anti–MDA5 antibody–positive DM is strongly associated with lethal RP-ILD and high mortality (7–11). Early intensive immunosuppressive therapy has been shown to markedly improve survival in these patients (12). In addition to pulmonary involvement, approximately 20% of patients with anti-MDA5 antibody–positive DM develop early liver dysfunction, which is also frequently observed in CADM (13). We previously demonstrated deposition of immunoglobulins and complement component C3 in lung tissues from patients with anti-MDA5 antibody–positive DM–associated ILD (14), suggesting a pathogenic role for antibody- and complement-mediated tissue injury in this disease.

CD206-positive M2-like macrophages, typically associated with tissue repair and anti-inflammatory responses, have recently been implicated in the pathogenesis of various diseases including autoimmune disorders, lung fibrosis and chronic pulmonary disorders (15, 16). Anti-MDA5 antibody-positive DM fatal cases, compared to surviving cases, have been reported to have increased serum levels of soluble CD206 proteins and increased expression of CD206-positive macrophages in lung tissue pathology with significant differences observed (17). These findings suggest that M2-like macrophages may play a pivotal role in mediating tissue injury and fibrosis in anti-MDA5 antibody–positive DM.

In the present study, we show that CD80-positive M1-like and CD206-positive M2-like macrophages are present in liver tissue from autopsy cases of DM with anti-MDA5 antibodies. Using human MDA5 transgenic (Tg) mice, we established a model of antibody-mediated hepatic injury by administering in-house anti-human MDA5 monoclonal antibodies (mAbs). This treatment induced hepatic infiltration by M2-like macrophages, whereas complement component C3–deficient MDA5 Tg mice were largely protected. Collectively, these findings implicate M2-like macrophages and complement activation as key mediators of hepatic injury in this model.

## Methods

### Human samples

Liver tissues were collected from 5 autopsies having DM at Kurume University, Hospital (Kurume, Japan), Nagasaki University Hospital (Nagasaki, Japan), and Hamamatsu University Hospital (Hamamatsu, Japan). The characteristics of autopsy cases examined for livers were shown in **Table 1**. Informed consent was obtained from all patients through an opt-out process.

**Table 1:** Characteristics of five autopsy cases examined for liver injury.

Case number	1	2	3	4	5
Sex	F	M	M	M	M
Age at death	69	52	59	53	64
Clinical diagnosis	DM	CADM	CADM	CADM	CADM
Clinical cause of death	ILD	ILD	ILD	Respiratory failure	ILD
Autopsy diagnosis	DAD	DAD	DAD	Undiagnosed	DAD
Onset to death (days)	64	768	83	72	87
ILD	+	+	+	+	+
Abdominal echo findings	Bright liver	ND	ND	ND	ND
HPS	–	–	–	–	–
Laboratory data at onset					
AST (U/L)	106	43	190	72	18
ALT (U/L)	63	28	228	106	12
LDH (U/L)	579	410	527	278	204
ALP (U/L)	301	205	128	417	252
γ-GT (U/L)	114	ND	84	497	25
CK (U/L)	1071	215	36	86	72
KL-6 (U/mL)	ND	2731	2922	1164	461
Ferritin (ng/mL)	2189	ND	1036	2783	260
ANA	Negative	160	Negative	Negative	Negative
MSA	anti–MDA5 Ab	anti–MDA5 Ab	anti–MDA5 Ab	anti–MDA5 Ab	anti–MDA5 Ab
MAA	Negative	Negative	ND	Negative	Negative
Treatment	mPSL, PSL, CyA, TAC	mPSL, PSL, CyA, TAC	mPSL, IVCY, TAC	PSL, TAC, IVCY	PSL, TAC, IVCY

M, male; F, female; CADM, clinically amyopathic dermatomyositis; DM, dermatomyositis; Ab, antibody; DAD, diffuse alveolar damage; ILD, interstitial lung disease; HPS, hemophagocytic syndrome; AST, aspartate aminotransferase; ALT, alanine aminotransferase; LDH, lactate dehydrogenase; ALP, alkaline phosphatase; γ-GT, γ-glutamyl transferase; CK, creatine kinase; KL-6, Krebs von den Lungen-6; ANA, antinuclear antibody; MSA, myositis-specific antibody; MAA, myositis-associated antibody; PSL, prednisolone; mPSL, methylprednisolone pulse therapy; CyA, cyclosporine A; TAC, tacrolimus; IVCY, intravenous cyclophosphamide; ND, not done.

### Establishment of in-house anti-human MDA5 antibodies

To generate anti-human MDA5 polyclonal antibody, specific pathogen-free (Japanese White) rabbits were immunized with recombinant human MDA5 protein. Purified antibody was generated from the antisera using a protein G column (Cytiva, Tokyo, Japan), as reported previously (14). We have reported the characterization of five mouse anti-human MDA5 mAbs clones [H5 (mouse IgG1), H27 (mouse IgG1), H46 (mouse IgG2b), H77 (mouse IgG2b), and H85 (mouse IgG1)] (14, 18), respectively.

### Animals

We generated three lines of the human MDA5 Tg mice (line no. 32, 55, and 116) (14). Complement component C3 deficient (-/-) mice were kindly obtained from Dr. Yoichiro Iwakura (Tokyo University of Science, Tokyo, Japan). We backcrossed line no. 55 of human MDA5 Tg mice with C3 (-/-) mice and generated the C3 (-/-) human MDA5 Tg mice. Genotyping was performed by PCR, as previously reported (19). Wild type male and female B6D2F1 (BDF1) mice were purchased from Charles River Japan (Yokohama, Japan). In this study, male and female Tg and wild type mice aged 8–12 weeks were used. Mice were anesthetized via intraperitoneal injection of medetomidine hydrochloride (0.3 mg/kg), midazolam (4 mg/kg), and butorphanol tartrate (5 mg/kg). Euthanasia was performed by cervical dislocation under deep anesthesia, which was confirmed by the absence of heartbeat and respiration.

### Anti-MDA5 antibody-induced hepatic injury mouse model

A mixture of purified anti-human MDA5 mAbs consisting of five clones (H5, H27, H46, H77, and H85) was prepared at a concentration of 0.1 mg per clone (total 0.5 mg) in 0.5 mL. Purified mouse IgG (Sigma-Aldrich, Tokyo, Japan) was used as a control antibody. Human MDA5 Tg and control wild type mice (n = 3 to 5 per group) were treated with 0.5 mg of the mAb mixture or control mouse IgG on days 0, 7, 14, and 21, and were sacrificed on day 28 for histological, single-cell RNA sequencing, and liver function analyses. Mice were also treated with 0.5 mL of rabbit anti-human MDA5 polyclonal antibodies (antisera) or normal rabbit serum four times (on days 0, 7, 14, and 21), as previously reported (14). At least three independent experiments were performed. Serum levels of AST, ALT, total bilirubin, total protein, and albumin on day 28 were measured at the Institute for Disease Modeling, Kurume University School of Medicine.

### Hematoxylin & eosin and immunohistochemistry staining

Tissues collected from mice were immersed in 10 % buffered formalin and stored at room temperature. On the following day, the fixative was replaced with fresh 10 % buffered formalin. Hematoxylin & eosin (H&E) staining were performed using deparaffinized sections. Immunohistochemistry staining was performed as we reported previously (14, 20, 21). Briefly, deparaffinization was carried out through cleansing with xylene and ethanol. Tissues were immersed in citrate buffer (10 mmol/L, pH 7.0) and subjected to antigen retrieval by autoclaving using the Pascal Decloaking Chamber (Dako, USA). Following antigen activation, tissues were incubated in 0.3% H_2_O_2_ for 10 min to block endogenous peroxidase activity. Primary antibodies [rabbit anti-mouse and human cytokeratin (CK) 8 + 18 mAb; 8C12 (catalog no. bsm-52419R, Bioss Inc., USA), rabbit anti-mouse F4/80 mAb; SP115 (catalog no. ab111101, Abcam, USA), rabbit anti-human and mouse CD80 polyclonal antibody (catalog no. ab254579, Abcam), rabbit anti-human and mouse mannose receptor, C type 1 (CD206) polyclonal antibody (catalog no. ab64693, Abcam), 1 to 4 μg/mL were utilized and incubated overnight at 4°C in a humid chamber. On the subsequent day, secondary antibodies (biotin-labeled goat anti-rabbit IgG antibody) were used, and color development was achieved using the Histofine DAB substrate kit (catalog no. 425011, Nichirei Biosciences Inc., Tokyo, Japan).

### Western blotting analysis

Western blot analysis was performed using protease inhibitors for serine and cysteine proteases (catalog no. 11836170001, Roche Diagnostics Deutschland GmbH, Germany), as previously reported (18, 22). Wild-type or complement component C3–deficient human MDA5 Tg mice (n = 5 per group) were treated with either an anti–human MDA5 mAb or control mouse IgG. Liver tissues were collected from individual mice in both the anti–human MDA5 mAb–treated and control IgG–treated groups and processed for western blot analysis.

Cell lysates were centrifuged, and equal amounts of protein (approximately 100 ng) were separated by SDS–PAGE using NuPAGE™ 4–12% Bis-Tris Mini Protein Gels (1.0–1.5 mm; catalog no. NP032, Thermo Fisher Scientific, USA) with NuPAGE^®^ MOPS SDS Running Buffer (catalog no. NP0001, Thermo Fisher Scientific, USA). Proteins were transferred to membranes using a dry blotting system (catalog no. IB1001, Invitrogen, USA). Membranes were blocked with Blocking One (catalog no. 03953-95, Nacalai Tesque, Kyoto, Japan) and incubated for 1 h at room temperature with rabbit anti-mouse F4/80 mAb (EPR26545-166; catalog no. ab300421, Abcam, USA; 1:500), rabbit anti-human and mouse CD80 polyclonal antibody (catalog no. ab254579, Abcam; 1:1000), rabbit anti-human and mouse mannose receptor, C type 1 (CD206) polyclonal antibody (catalog no. ab64693, Abcam; 1:1000), and mouse anti–β-actin mAb (AC-15; catalog no. A5441, Sigma-Aldrich, USA; 1:5,000). After washing, membranes were incubated with horseradish peroxidase (HRP)–conjugated goat anti-rabbit IgG (H+L) (catalog no. 5220-0336, Kirkegaard & Perry Laboratories, Inc., USA; 1:5000) and/or HRP-conjugated goat anti-mouse IgG (H+L) (catalog no. 330, Medical & Biological Laboratories Co., Ltd., Tokyo, Japan; 1:5,000). Signals were detected using SuperSignal™ West Pico PLUS Chemiluminescent Substrate (catalog no. 34577, Thermo Fisher Scientific, USA) and visualized with an Amersham™ ImageQuant™ 800 system (Cytiva, Tokyo, Japan). Band intensities were quantified using ImageJ software (National Institutes of Health, Bethesda, MD, USA), and protein expression levels were normalized to β-actin.

### Single-cell RNA-sequencing library preparation from frozen mouse livers

For single-cell RNA sequencing, approximately 25 mg of liver tissue obtained from three individual mice in the anti-human MDA5 mAb-treated group and three mice in the control IgG-treated group were dissociated and pooled separately prior to library preparation, generating one mixed sample per group (total of 75 mg per group). Two independent experiments were performed. Pooling was performed to increase biological representativeness by reducing inter-individual variability and to enhance the detection of rare cell populations. Because each group yielded a single pooled library, biological replicates were not available for statistical comparisons at the single-cell level. Consequently, group and sample are completely confounded, and formal statistical testing or false discovery rate (FDR)–based multiple-testing correction for differentially expressed genes (DEGs) is not valid or interpretable for this dataset.

Snap-frozen mouse liver tissues were processed at Takara Bio Inc. (Tokyo, Japan) using a gentleMACS Dissociator (Miltenyi Biotec, Tokyo, Japan) in combination with the Chromium Next GEM Single Cell Fixed RNA Sample Preparation Kit (10x Genomics, Pleasanton, CA, USA). A total of 2 × 10^6^ cells were hybridized with barcoded probes, pooled, and encapsulated using a Chromium X instrument with the Chromium Fixed RNA Kit, Mouse Transcriptome, 4 rxns × 4 BC, and Next GEM Chip Q. After primer extension and cDNA amplification, libraries were sequenced on an Illumina platform.

Sequencing data were processed using the default Cell Ranger pipeline (10x Genomics), including barcode filtering, read alignment, and cell calling. No additional quality-control procedures, such as dead-cell removal, doublet detection/removal, or ambient RNA decontamination, were applied. Downstream analysis and visualization were performed using Loupe Browser v9.0.0. Dimensionality reduction was conducted by principal component analysis (PCA; first 30 principal components), followed by graph-based clustering using the Louvain algorithm. t-distributed stochastic neighbor embedding (t-SNE) was used for two-dimensional visualization.

Major cell clusters were annotated based on canonical marker genes, including hepatocytes (Hep), cholangiocytes/hepatic progenitor cells (Cho/HPCs), endothelial cells (Endo), hepatic stellate cells (HSCs), Kupffer cells (KCs), myofibroblasts (Myo), monocytes or monocyte-derived macrophages (Mo/MoMFs), dendritic cells (DCs), plasmacytoid DCs (pDCs), T/NK cells (T/NK), neutrophils (N), and B cells (B), as previously reported (22).

In this study, cells expressing *Adgre1* (F4/80) were annotated as macrophage-lineage cells. Cells co-expressing *Adgre1* (F4/80) and *Cd86* or *Adgre1* (F4/80) and *Cd163* were operationally classified as M1-like and M2-like macrophages, respectively. This gene-based annotation enabled descriptive assessment of heterogeneous macrophage activation states within liver tissue.

To assess complement activation, the expression of *C1s1*, *C3*, and *C4b* was examined, as previously reported (23).

### Statistical analysis

Differences in serum biochemical parameters among the four groups of mice were analyzed using one-way analysis of variance (ANOVA). When a significant main effect was detected, *post hoc* comparisons were performed using the Tukey–Kramer test. Statistical analyses for western blotting were performed using the Mann–Whitney U test. For comparisons among the four experimental groups (n = 6 biological replicates per group), pairwise comparisons were conducted as indicated in the figure legends. Data are presented as mean ± standard error of the mean (SEM). Statistical analyses were conducted using JMP^®^ Student Edition 18.2.1 (SAS Institute, Cary, NC, USA). A p value < 0.05 was considered statistically significant.

## Results

### CD206 positive macrophages in livers of patients with anti-MDA5 autoantibody positive DM

We obtained liver tissues from five anti-MDA5 antibodies positive DM autopsy cases of DM. Four of the five anti-MDA5–positive DM patients were diagnosed with clinically amyopathic dermatomyositis (CADM). Serum levels of AST and or ALT were increased in 4 of 5 autopsied patients (**Table 1**). A previous study reported that liver biopsy specimens from DM patients positive for anti-MDA5 antibodies showed steatosis, hepatocellular ballooning, increased numbers of pigmented macrophages, and glycogenated nuclei (13, 24). In contrast, hepatic architecture, including the sinusoidal structure, was preserved, and no hepatocellular destruction was found. There were inflammatory cells in H&E staining and Immunohistochemical analysis was subsequently performed. Anti–CK 8 + 18 mAb were used to assess hepatocyte structural integrity, and anti-F4/80 mAb were used to identify myeloid-lineage cells. Anti-CD80 polyclonal antibodies were used to identify M1-like macrophages, and anti-CD206 polyclonal antibodies were used to identify M2-like macrophages.

**Figure 1:**
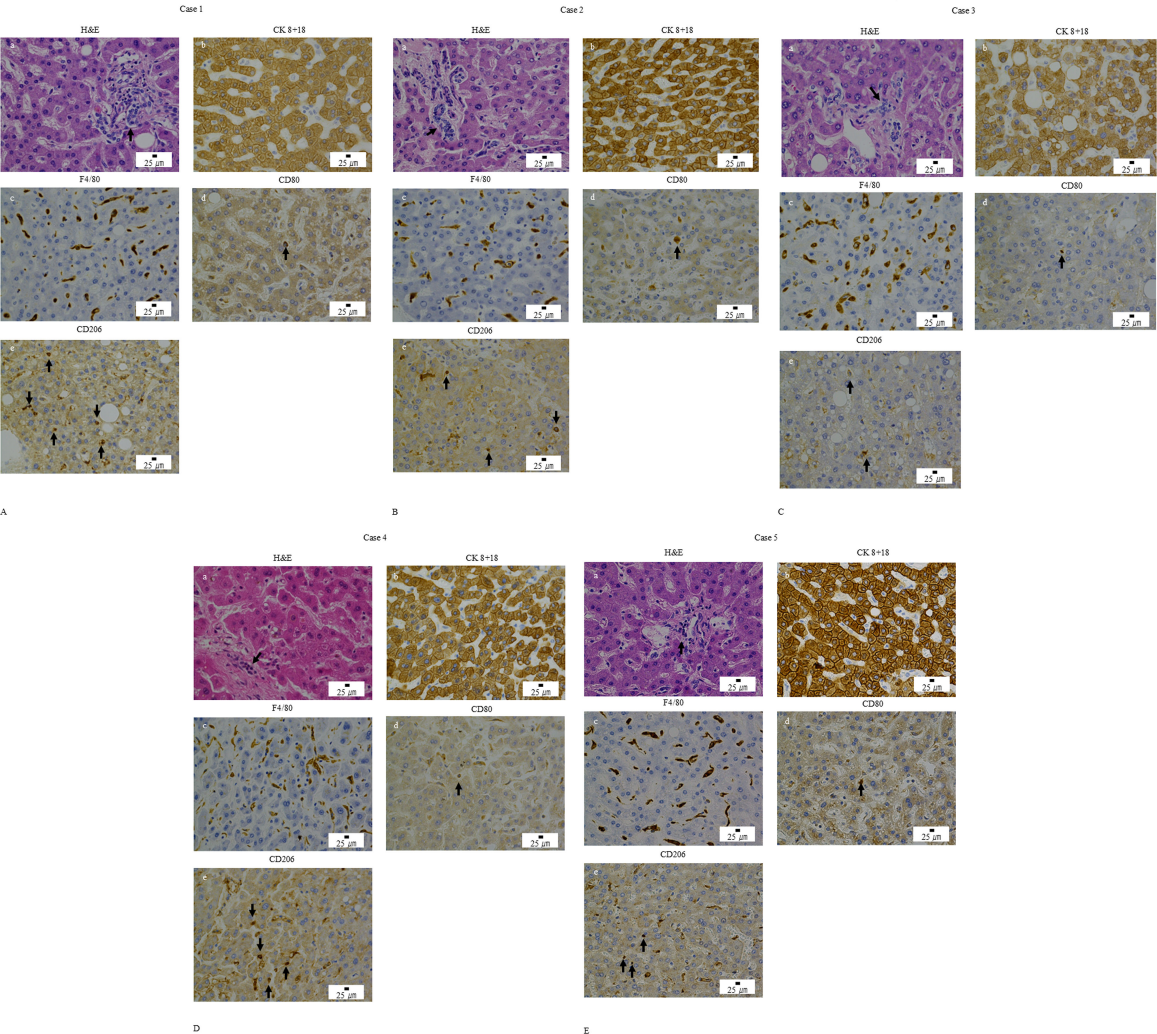
Histopathological findings of liver tissues from five autopsy cases of anti–MDA5 antibody–positive dermatomyositis. Representative histological images from autopsy case 1 **(A)**, case 2 **(B)**, case 3 **(C)**, case 4 **(D)**, and case 5 **(E)** are shown. (a) Hematoxylin and eosin (H&E) staining demonstrating inflammatory cell infiltration (arrows). (b) Immunohistochemical staining for cytokeratin 8 + 18. (c) Immunohistochemical staining for F4/80. (d) Immunohistochemical staining for CD80, with CD80-positive cells indicated (arrows). (e) Immunohistochemical staining for CD206, with CD206-positive cells indicated (arrows).

Immunostaining for CK8/18 showed preserved expression patterns in hepatocytes. F4/80-positive cells were observed in liver tissues. CD206-positive macrophage-like cells were readily detectable, whereas CD80-positive macrophage-like cells were less frequently observed. Notably, both CD80-positive and CD206-positive macrophages were detected in the livers of all five anti–MDA5 autoantibody–positive DM cases (**Figures 1A–E**, corresponding to cases 1–5, respectively). These findings indicate the presence of macrophage populations expressing either CD80 and/or CD206 in the affected livers.

### Anti-human MDA5 antibodies induce hepatic injury in human MDA5 transgenic mice

We examined whether anti-human MDA5 antibody can induce hepatic injury and M2-like macrophage inflammation in human MDA5 Tg mice. In this study, human MDA5 Tg mice (line 55) treated with anti-human MDA5 polyclonal antibody exhibited more liver and lung injury accompanied by inflammatory cell infiltrates than either untreated mice or those receiving control rabbit serum (**Supplementary Figure S1**). Next, we treated all three Tg mouse lines (Nos. 32, 55, and 116) with the anti-human MDA5 polyclonal antibody. We observed hepatic injury, characterized by hepatocellular swelling, disruption of the normal sinusoidal architecture, and focal lymphocytic infiltration in lines 32 and 55, but pathology was normal in line 116 (**Supplementary Figure S2**).

**Figure 2:**
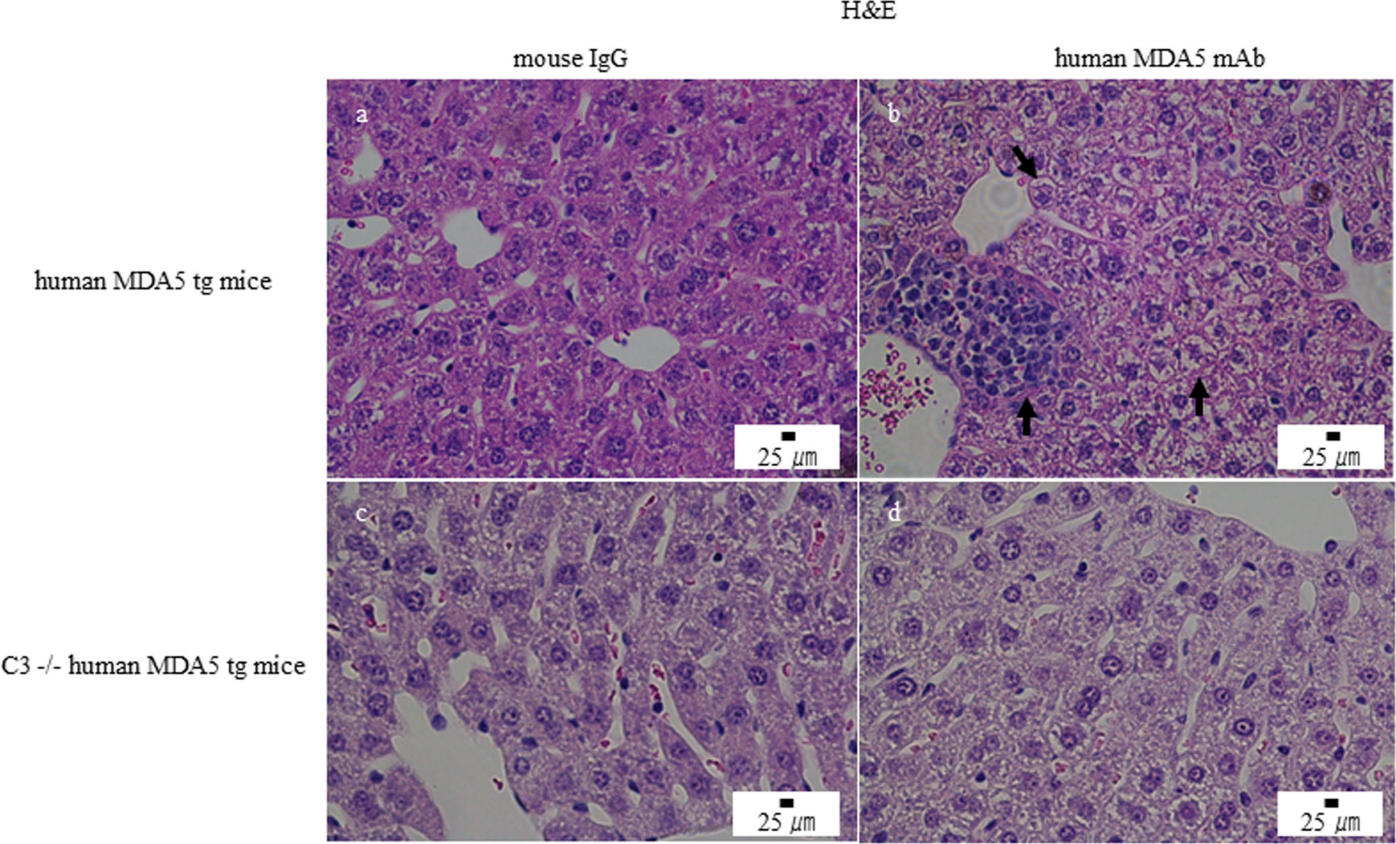
Anti–human MDA5 monoclonal antibody treatment induces hepatic injury in human MDA5 transgenic mice but not in complement component C3–deficient human MDA5 transgenic mice. Human MDA5 Tg mice were administered 0.5 mg of control mouse IgG **(a, c)** or an anti–human MDA5 mAb mixture **(b, d)** on days 0, 7, 14, and 21, and were sacrificed on day 28. Representative hematoxylin and eosin (H&E)–stained liver sections are shown. **(a, b)** Wild-type human MDA5 Tg mice. **(c, d)** Complement component C3–deficient human MDA5 Tg mice. Arrows indicate areas of inflammatory cell infiltration and hepatic injury.

**Figure 3:**
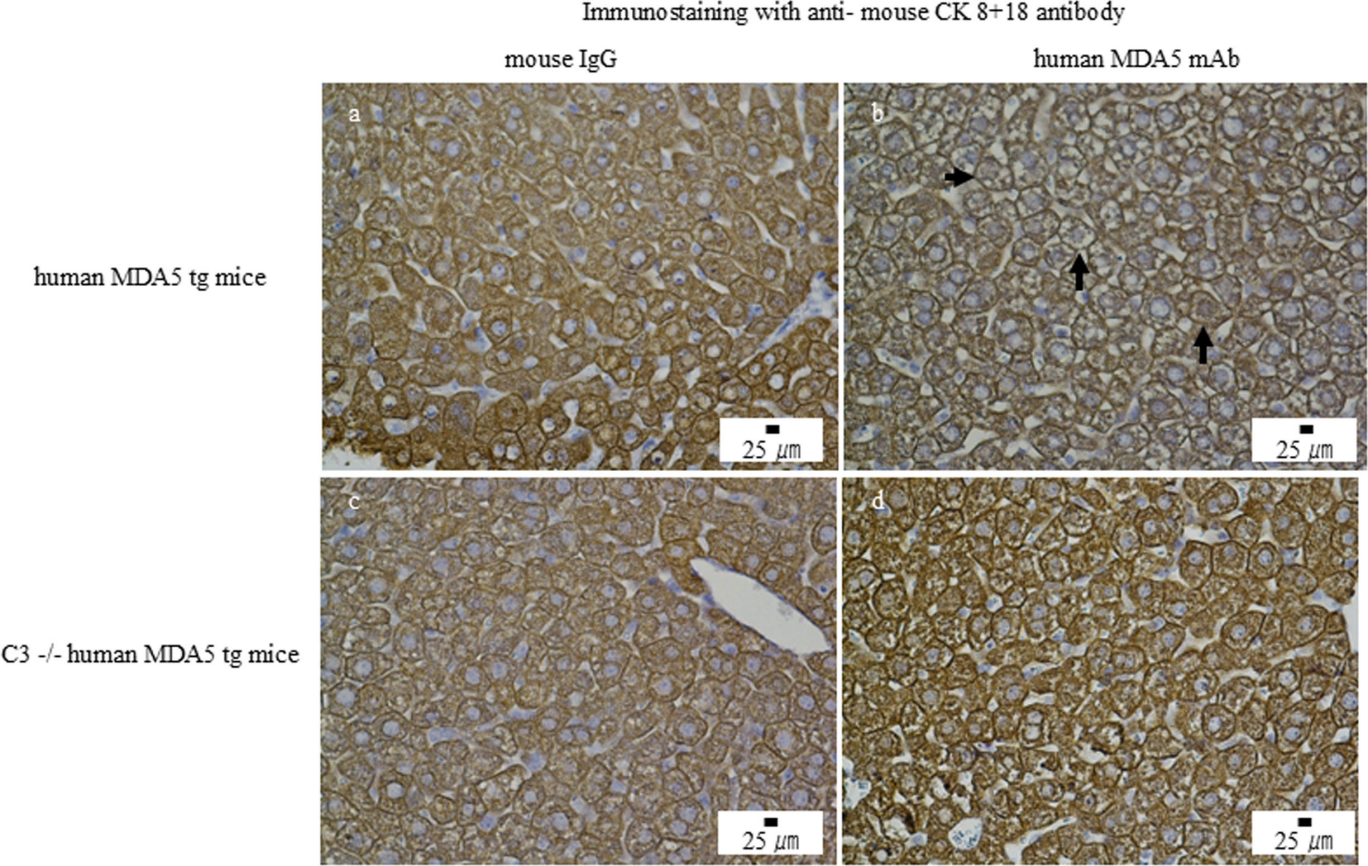
Anti–human MDA5 monoclonal antibody treatment induces hepatic injury in human MDA5 transgenic mice but not in complement component C3–deficient human MDA5 transgenic mice. Human MDA5 Tg mice were administered 0.5 mg of control mouse IgG **(a, c)** or an anti–human MDA5 mAb mixture **(b, d)** on days 0, 7, 14, and 21, and were sacrificed on day 28. Representative liver sections stained for cytokeratin 8 + 18 are shown. **(a, b)** Wild-type human MDA5 Tg mice. **(c, d)** Complement component C3–deficient human MDA5 Tg mice. Arrows indicate areas of hepatocellular injury.

We then administered five different anti-human MDA5 mAb clones (H5, H27, H46, H77, and H85) to Tg line 55 mice. Each clone induced hepatic injury with inflammatory infiltrates, whereas control IgG induced only minimal injury (**Supplementary Figure S3**). Based on this finding, we treated Tg mice with either a mixture of all five mAbs (0.1 mg per clone; total 0.5 mg) or control IgG on days 0, 7, 14, and 21. Histological analysis on day 28 revealed only minimal liver changes in control IgG–treated mice, whereas mAb mixture–treated mice showed pronounced hepatic injury with hepatocyte swelling, sinusoidal collapse, and lymphocytic infiltration (**Figure 2**). CK 8 + 18 expression was preserved in control IgG–treated mice but reduced in hepatocytes from mAb mixture–treated mice (**Figure 3**). Interestingly, both groups displayed only minimal lung injury (**Supplementary Figure S4**). We additionally examined the effects of anti-human MDA5 mAb treatment on the ear skin of human MDA5 Tg mice and observed no significant histological abnormalities (**Supplementary Figure S5**).

### Reduced hepatic injury in complement component C3-deficient human MDA5 transgenic mice

To clarify the role of complement, we generated C3 (-/-) human MDA5 Tg mice. C3 (+/+) wild type and C3 (-/-) Tg mice were treated with either control IgG or the anti-MDA5 mAb mixture. H&E staining showed that hepatic injury was markedly attenuated in C3 (-/-) Tg mice compared with C3 (+/+) Tg mice (**Figure 2**). Consistently, CK 8 + 18 expression was preserved in C3 (-/-) mice but reduced in C3 (+/+) mice (**Figure 3**). These findings indicate that complement activation contributes to anti-MDA5 antibody–induced hepatic injury.

Serum biochemical analyses (AST, ALT, total bilirubin, total protein, and albumin) revealed no significant differences between mAb mixture– and control IgG–treated groups, regardless of complement status (**Supplementary Figure S6**).

### Increased M2-like macrophages in the livers of human MDA5 transgenic mice

**Figure 4:**
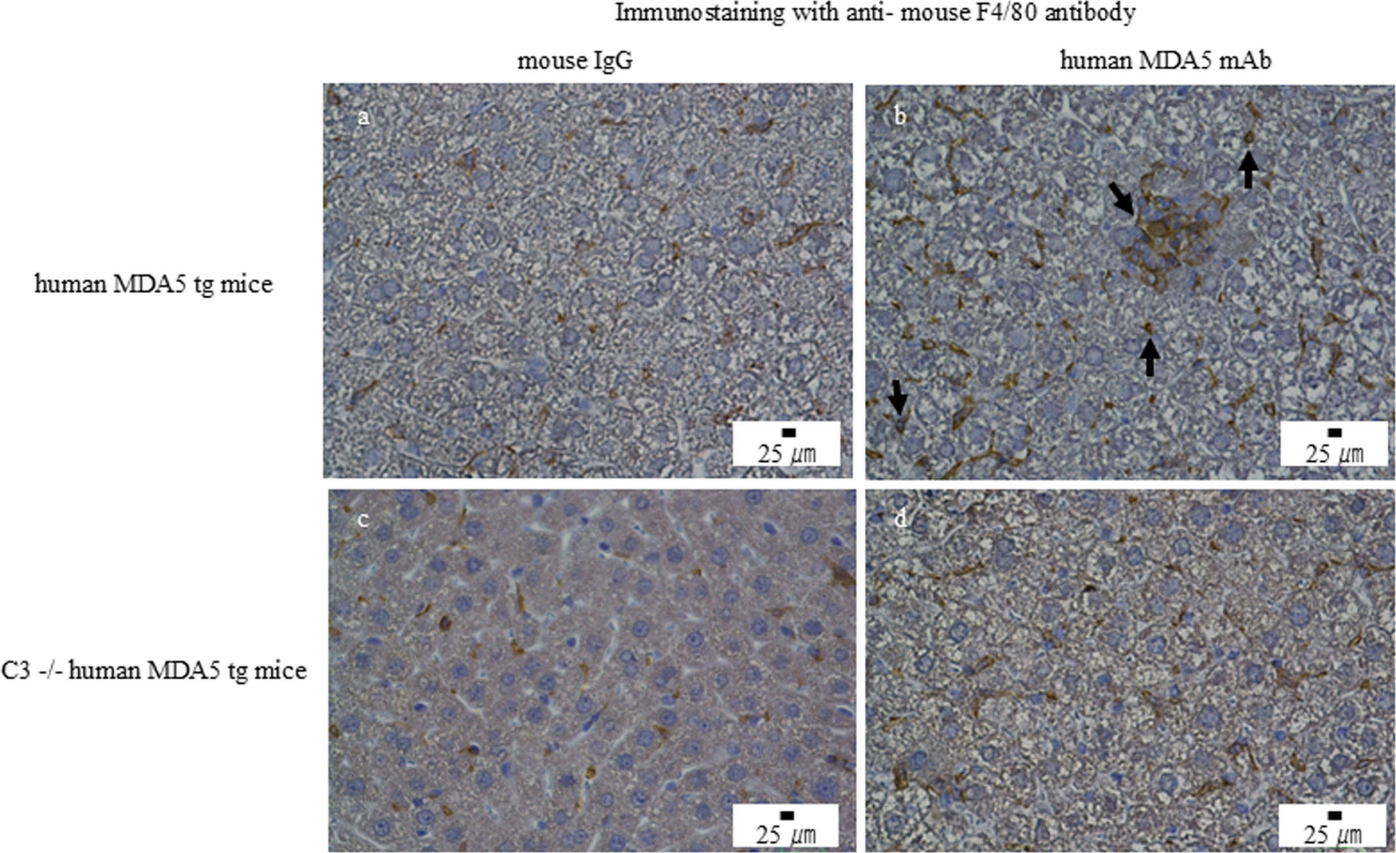
Anti–human MDA5 monoclonal antibody treatment is associated with increased F4/80-positive macrophages in the liver of human MDA5 transgenic mice but not in complement component C3–deficient mice. Human MDA5 Tg mice were administered 0.5 mg of control mouse IgG **(a, c)** or an anti–human MDA5 mAb mixture **(b, d)** on days 0, 7, 14, and 21, and were sacrificed on day 28. Representative liver sections stained for F4/80 are shown. **(a, b)** Wild-type human MDA5 Tg mice. **(c, d)** Complement component C3–deficient human MDA5 Tg mice. Arrows indicate areas with increased F4/80-positive macrophages.

**Figure 5:**
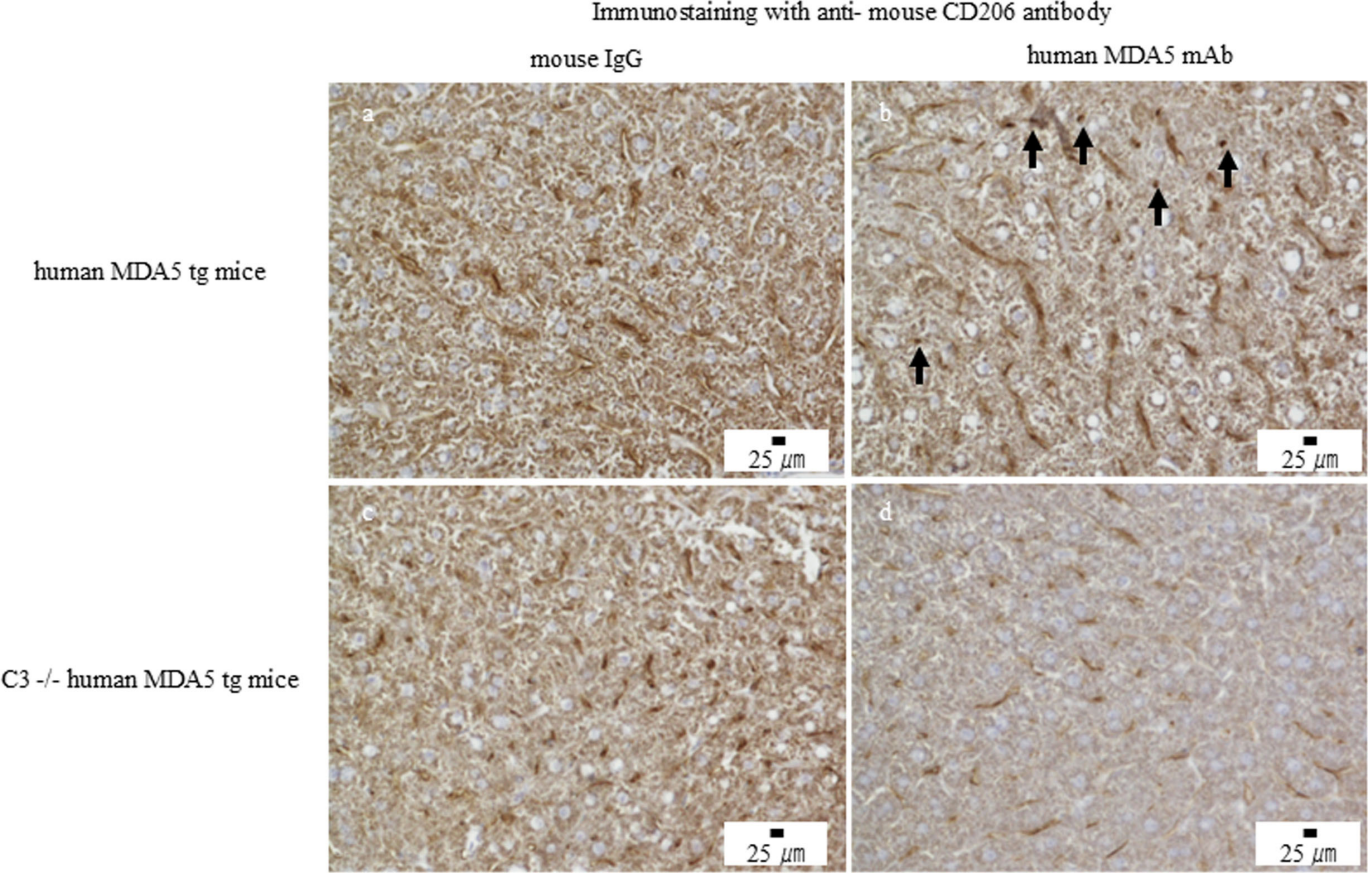
Anti–human MDA5 monoclonal antibody treatment is associated with increased CD206-positive macrophages in the liver of human MDA5 transgenic mice but not in complement component C3–deficient mice. Human MDA5 Tg mice were administered 0.5 mg of control mouse IgG **(a, c)** or an anti–human MDA5 mAb mixture **(b, d)** on days 0, 7, 14, and 21, and were sacrificed on day 28. Representative liver sections stained for CD206 are shown. **(a, b)** Wild-type human MDA5 Tg mice. **(c, d)** Complement component C3–deficient human MDA5 Tg mice. Arrows indicate CD206-positive macrophages.

**Figure 6:**
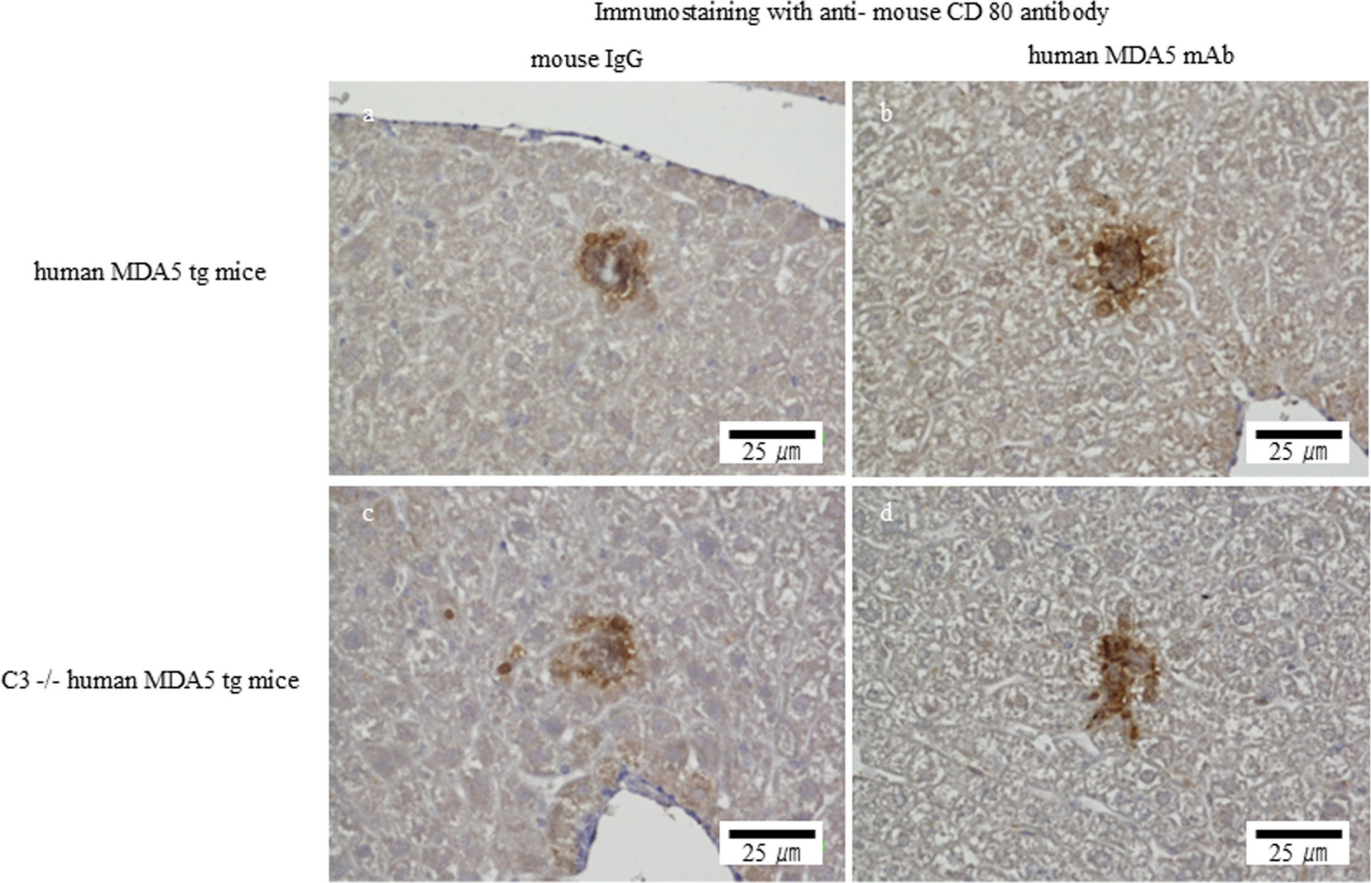
Anti–human MDA5 monoclonal antibody treatment is not associated with an apparent increase in CD80-positive macrophages in the liver of human MDA5 transgenic mice. Human MDA5 Tg mice were administered 0.5 mg of control mouse IgG **(a, c)** or an anti–human MDA5 mAb mixture **(b, d)** on days 0, 7, 14, and 21, and were sacrificed on day 28. Representative liver sections stained for CD80 are shown. **(a, b)** Wild-type human MDA5 Tg mice. **(c, d)** Complement component C3–deficient human MDA5 Tg mice.

Immunohistochemistry confirmed increased F4/80-positive macrophages in the livers of Tg mice treated with the mAb mixture compared with controls (**Figure 4**). Notably, CD206-positive M2-like macrophages were increased in the mAb-treated group (**Figure 5**), whereas CD80-positive M1-like macrophages were scarcely detected (**Figure 6**). Importantly, CD206-positive M2-like macrophage infiltration was reduced in C3 (-/-) Tg mice treated with the mAb mixture (**Figure 5**).

To enable semi-quantitative evaluation, western blot analysis was performed. Human MDA5 Tg mice and C3 (-/-) human MDA5 Tg mice were treated with either an anti-human MDA5 mAb mixture or control mouse IgG (six mice per group). Livers were isolated from individual mice, and three independent experiments were conducted. Hepatic F4/80 protein levels were significantly higher (p < 0.05) in human MDA5 Tg mice treated with the anti-human MDA5 mAb mixture compared with those treated with control mouse IgG. A significant elevation in hepatic F4/80 protein levels (p < 0.05) was also observed in C3-sufficient (C3 +/+) human MDA5 Tg mice treated with the anti-human. MDA5 mAb mixture compared with identically treated C3 (-/-) human MDA5 Tg mice. In contrast, no significant differences in F4/80 protein levels were detected between control mouse IgG–treated and anti-human.

**Figure 7:**
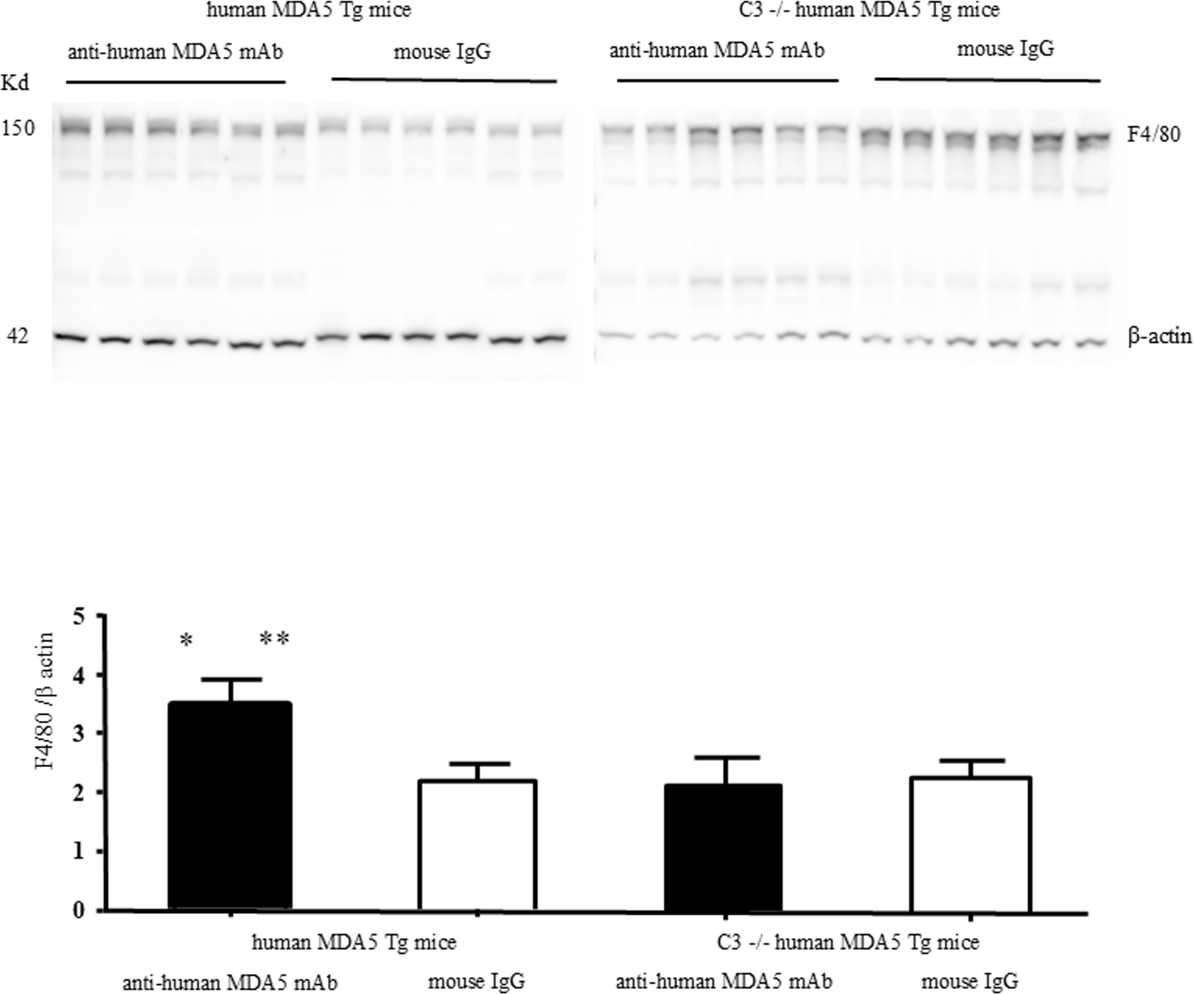
Western blot analysis of liver tissues from human MDA5 transgenic mice with or without C3 deficiency. Representative western blot images of liver tissues from human MDA5 Tg mice and C3-deficient (C3^−^/^−^) human MDA5 Tg mice treated with either anti-human MDA5 mAb or control mouse IgG are shown. The left panel shows human MDA5 Tg mice, and the right panel shows C3^−^/^−^ human MDA5 Tg mice. F4/80 expression was analyzed, with β-actin used as a loading control. Each panel shows independent biological replicates. Quantitative analysis was performed by normalizing F4/80 expression to β-actin (expressed as the F4/80/β-actin ratio), as described in the Methods. Data are presented as mean ± SEM. Statistical significance was determined using the Mann–Whitney U test. Pairwise comparisons among the experimental groups were performed as indicated (n = 6 biological replicates per group). *P < 0.05 vs. control mouse IgG–treated human MDA5 Tg mice. **P < 0.05 vs. anti-human MDA5 mAb–treated C3^−^/^−^ human MDA5 Tg mice.

MDA5 mAb–treated C3 (-/-) human MDA5 Tg mice. Furthermore, no significant differences were observed between control mouse IgG–treated C3 (+/+) and C3 (-/-) human MDA5 Tg mice (**Figure 7**). Because quantitative evaluation of CD206 immunostaining on tissue sections was technically challenging due to heterogeneous staining patterns, we attempted to perform western blot analyses for CD80 and CD206, as conducted for F4/80. However, despite repeated experiments, CD80 and CD206 protein levels were not consistently detectable by western blotting, precluding reliable quantitative analysis (**Supplementary Figure S7**). Therefore, these data were not suitable for quantitative comparisons.

### Single-cell RNA sequencing identifies macrophage clusters with M2-associated gene expression in the livers of human MDA5 Tg mice

**Figure 8:**
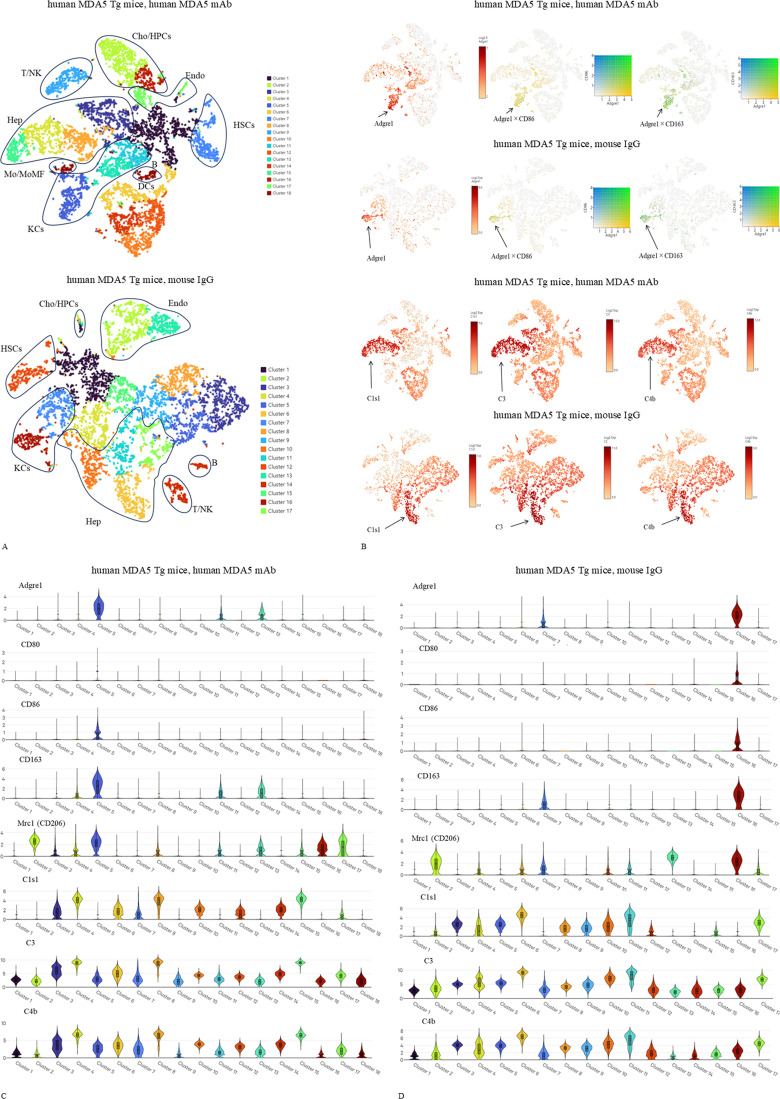
Single-cell RNA sequencing analysis reveals macrophage populations in the livers of anti–human MDA5 antibody–treated mice. Female human MDA5 Tg mice (n = 5 per group) were injected intraperitoneally with 0.5 mg of an anti–human MDA5 mAb mixture or control mouse IgG on days 0, 7, 14, and 21, and were sacrificed on day 28. Liver tissues from three of the five mice per group were snap-frozen and used for single-cell RNA sequencing. Two independent pooled experiments were performed. Data analysis was conducted using Loupe Browser v9.0.0 (10x Genomics). Clustering was performed using the default graph-based Louvain algorithm implemented in Loupe Browser and visualized by t-distributed stochastic neighbor embedding (t-SNE). **(A)** Full cell-type annotations of liver cells from human MDA5 Tg mice treated with anti–human MDA5 mAb or control mouse IgG. Clusters were annotated based on canonical marker genes and reference-based annotation, as previously reported (22). **(B)** Feature plots showing the expression of *Adgre1*, *C1s1*, *C3*, and *C4b*, as well as the co-expression of Adgre1 with *Cd86* or *Cd163*, displayed on t-SNE maps from the livers of human MDA5 Tg mice treated with anti–human MDA5 mAb or control mouse IgG. **(C)** Violin plots showing the expression levels of *Adgre1* (F4/80), *Cd80*, *Cd86*, *Cd163*, *Mrc1* (CD206), *C1s1*, *C3*, and *C4b* across 18 clusters in the livers of human MDA5 Tg mice treated with anti–human MDA5 mAb. Gene expression levels are shown as log2-normalized values. **(D)** Violin plots showing the expression levels of *Adgre1* (F4/80), *Cd80*, *Cd86*, *Cd163*, *Mrc1* (CD206), *C1s1*, *C3*, and *C4b* across 17 clusters in the livers of human MDA5 Tg mice treated with control mouse IgG. Gene expression levels are shown as log2-normalized values.

We performed single-cell RNA sequencing of liver tissues from human MDA5 Tg mice treated with either an anti–human MDA5 mAb mixture or control mouse IgG. Major cell clusters were annotated based on canonical marker genes, as previously reported (22) (**Figure 8A**). In anti–human MDA5 mAb–treated mice, we identified a distinct macrophage cluster (cluster 5) expressing *Adgre1* (F4/80), together with macrophage-associated genes including *Lilrb4a*, *Tbxas1*, *Fgd2*, and *Ccr5*, consistent with prior reports (16) (**Table 2**). In control mouse IgG–treated mice, a macrophage cluster (cluster 16) expressing *Adgre1* and the same set of macrophage-associated genes (*Lilrb4a*, *Tbxas1*, *Fgd2*, and *Ccr5*) was also identified (**Table 3**).

**Table 2:** Table summarizes the top 20 genes with the highest expression in cluster 5 from the livers of human MDA5 transgenic mice treated with anti-human MDA5 monoclonal antibodies.

Gene name	Cluster 5 average	Cluster 5 Log2 fold change	Cluster 5 P-value
Lilrb4a	1.409392248	6.556867012	3.20E-255
Tbxas1	1.915892588	6.490982042	1.15E-314
Fgd2	1.319103058	6.345607438	1.79E-231
Lair1	1.02180938	6.248828302	3.87E-211
Slc8a1	1.491973825	6.232877083	1.20E-237
Themis2	2.183456897	6.211650601	4.19E-287
Lilra5	1.018506117	6.197658797	2.14E-209
Igsf6	1.168254043	6.16029121	1.40E-213
Slc11a1	2.443313593	6.103404586	8.35E-276
Dmpk	9.02011039	6.093898579	1.12E-280
Hk3	1.123109448	6.057175117	1.67E-201
Adgre4	1.225510604	6.002485814	3.71E-203
Ccr5	1.756234872	5.990279022	5.37E-252
Pla2g7	1.364247653	5.958106396	8.63E-235
Siglec1	2.506075592	5.958078965	2.39E-255
Lrrc25	1.577858666	5.955684104	2.59E-248
Cd163	5.621052678	5.955334888	3.53E-257
Pirb	1.969845885	5.945539737	5.18E-249
Gbp2b	2.742809446	5.919338067	3.57E-253
Oas2	1.531612982	5.914114019	1.54E-240

The values represent relative normalized expression levels set for convenience.

**Table 3:** Table summarizes the top 20 genes with the highest expression in cluster 16 from the livers of human MDA5 transgenic mice treated with control mouse IgG.

Gene name	Cluster 16 average	Cluster 16 Log2 fold change	Cluster 16 P-value
Dmpk	16.30753162	6.549563765	2.33E-152
Siglec1	3.936300736	6.668627266	8.90E-152
Clec4f	35.09154383	6.435942516	4.39E-150
Slc11a1	3.496865081	6.592463505	7.92E-148
Adgre1	4.658762744	6.469597013	1.74E-140
Cd163	7.947082091	6.450383339	4.54E-139
Slc8a1	2.565857339	6.934063401	6.31E-137
Fgd2	2.174834087	7.05246876	8.27E-136
Tbxas1	2.655234082	6.886666341	4.97E-135
Oas2	3.340455781	6.720471047	1.22E-134
Themis2	2.733438732	6.875315858	6.87E-131
Lilrb4a	2.11152556	7.050950333	3.86E-129
C6	4.748139487	6.243098513	3.95E-126
Ccr5	2.316347264	6.694906726	4.03E-125
Csf1r	9.969230908	6.133714794	8.15E-125
Pirb	2.521168967	6.64923239	9.14E-124
Igsf6	1.899255795	6.933486496	1.91E-121
Fyb	3.22501082	6.465198711	2.39E-120
Gbp2b	2.595649586	6.587246711	3.04E-120
Cd300a	2.941984467	6.439643171	2.98E-118

The values represent relative normalized expression levels set for convenience.

Feature plots illustrate enhanced expression of *Adgre1*, as well as co-expression of *Adgre1* with *Cd86* or *Cd163*, in the livers of anti–human MDA5 mAb–treated human MDA5 Tg mice compared with control mouse IgG–treated human MDA5 Tg mice (**Figure 8B**). We next examined complement-related gene expression. As previously reported (23), the expression of *C1s1*, *C3*, and *C4b* was assessed across cell clusters. Notably, transcripts for *C1s1*, *C3*, and *C4b* were broadly detected across multiple cell clusters in the livers of human MDA5 Tg mice treated with either the anti–human MDA5 mAb mixture or control mouse IgG (**Figure 8B**).

Violin plots show the expression levels of *Adgre1* (F4/80), *Cd80*, *Cd86*, *Cd163*, *Mrc1* (CD206), *C1s1*, *C3*, and *C4b* across 18 clusters in the livers of anti–human MDA5 mAb–treated human MDA5 Tg mice (**Figure 8C**). These macrophages exhibited increased expression of M2-associated polarization markers *Cd163* and *Mrc1*, while also expressing the M1-associated costimulatory molecules *Cd80* and *Cd86*. In contrast, macrophages identified in control mouse IgG–treated mice showed substantially lower expression of *Cd163* and *Mrc1*, as well as *Cd80* and *Cd86* (**Figure 8D**). Taken together, these findings indicate increased expression of macrophage-associated genes, including M2-related markers, in the livers of human MDA5 Tg mice treated with anti–human MDA5 mAb, based on descriptive single-cell transcriptomic analysis.

## Discussion

It has been reported that in muscle tissues from patients with DM, immunoglobulin and complement deposits accumulate characteristically within intramuscular arterioles and capillaries, and this immune-complex deposition is responsible for the microangiopathy that typifies the disorders (25). In DM skin lesions, membrane attack complex (MAC) deposition was found in 86% of patients along the dermo-epidermal junction and in 77% of patients in the dermal vessel walls (26). In atrophic areas of muscle tissue, immune complexes are deposited in blood vessels (27). Lahoria et al. reported that the presence of C1q, C4d, and occasional IgM in MAC-positive capillaries and capillary remnants in muscle tissue of DM patients providing strong evidence for activation of the classical complement pathway (28). We have reported predominant deposition of C3, IgG, and IgM in the lung tissues from patients with anti-MDA5 antibody–positive DM-associated ILD, compared with patients with idiopathic pulmonary fibrosis (14). In this study, hepatic injury was observed in human MDA5 Tg mice treated with an anti-human MDA5 mAbs mixture, but not in MDA5 Tg mice lacking complement component C3 that received the same treatment. These findings suggest that complement activation contributes to the hepatic injury seen in our mouse model.

The experimental antibody-induced liver injury model used in this study was designed to isolate the direct effects of anti–human MDA5 antibodies and complement activation on hepatic inflammation. In this model, exogenous anti–human MDA5 mAbs were administered at a relatively high dose, resulting in an acute and complement-dependent inflammatory response that predominantly affected internal organs such as the liver and lung. No DM-like cutaneous manifestations were observed in anti–human MDA5 mAb–treated human MDA5 Tg mice. In patients with DM, characteristic skin rashes and muscle involvement are thought to arise from chronic immune activation, sustained type I interferon signaling, and microvascular injury, processes (24) that are not fully reproduced by short-term exogenous antibody administration. Accordingly, the present experimental system should be interpreted as a liver-predominant, antibody–complement–mediated injury model intended for mechanistic investigation rather than as a phenocopy of the full systemic spectrum of DM. While this reductionist approach allows focused analysis of antibody–complement–macrophage interactions, it also represents an important limitation. Future studies employing longitudinal designs, endogenous autoantibody models, or tissue-specific approaches will be required to more faithfully recapitulate muscle and skin involvement and to establish causal relationships in the pathogenesis of anti–MDA5 antibody–positive DM.

In this study, we showed anti-human MDA5 polyclonal antibodies induce severe lung injury as well as hepatic injury in human MDA5 Tg mice. In contrast, administration of the anti-human MDA5 mAbs mixture used in this study resulted in minimal pulmonary injury, but hepatic injury in human MDA5 Tg mice. Several reasons may account for this difference: (1) Differences in immune complex formation capacity between polyclonal and monoclonal antibodies. Polyclonal antibodies can bind to multiple epitopes, and even when target antigens are expressed at low levels on the cell surface, numerous antibody–antigen interactions facilitate the formation of large immune complexes. Deposition of these complexes in the lungs can trigger complement activation and a strong inflammatory response, leading to diffuse lung injury. (2) Differences in complement production, MDA5 expression, and immune complex clearance between liver and lung. The liver can serve as both a major site of complement production and MDA5 expression, and a central organ for immune complex clearance. (3) Differences in antibody isotype and complement-activating capacity. The polyclonal antibodies used in this study were rabbit IgG, which can activate the murine complement system. In contrast, mAbs used here (derived from mouse IgG1 or IgG2b) can exhibit reduced ability to activate murine complement. As a result, although hepatic injury was induced by anti-human MDA5 mAbs, the complement cascade was not sufficiently engaged to cause diffuse lung damage. Further analysis should be needed to clarify this issue.

It has been reported that, under physiological (steady-state) conditions, M1 and M2 inflammatory macrophages are absent or only minimally detectable in healthy human liver, whereas macrophage accumulation and polarization become evident mainly in inflammatory or pathological conditions (29, 30). In autopsy liver tissues from anti–MDA5 antibody–positive DM patients, hepatocellular destruction was less pronounced than that reported in a prior liver biopsy study (13). This discrepancy may reflect the fact that autopsy specimens represent the terminal stage of the disease. It is also possible that aggressive immunosuppressive therapy administered before death mitigated hepatic injury. These considerations highlight the importance of incorporating temporal context when interpreting tissue pathology. In the present study, CD80-positive M1-like and CD206-positive M2-like macrophages were observed in the autopsy livers of all five anti–MDA5 antibody–positive DM patients. However, this observation alone does not establish whether these macrophages act as pathogenic drivers, secondary responders to hepatic injury, or nonspecific features of end-stage disease. Because autopsy tissues reflect the final phase of illness, temporal relationships and causal roles cannot be inferred from these samples.

As we described above, although our autopsy specimens showed relatively modest hepatocyte destruction compared with previously reported liver biopsy findings (13), published data on liver pathology in adult patients with anti–MDA5 antibody–positive DM remain extremely limited. To our knowledge, only a small number of liver biopsy cases have been reported in this population, including four patients described in prior studies (13, 24). While treatment effects or advanced disease stage may contribute to the observed differences between autopsy and biopsy findings, such interpretations remain speculative in the absence of longitudinal or early-stage liver samples. Future studies incorporating serial tissue sampling or mechanistic approaches, such as targeted macrophage depletion, will be required to clarify the temporal and pathogenic significance of CD80-positive M1-like and CD206-positive M2-like macrophage infiltration.

In this study, we observed the presence of CD206-positive M2-like macrophages in the livers of anti–human MDA5 mAb–treated mice, in association with hepatic injury. At first glance, these findings appear to contrast with the conventional paradigm in which M2 macrophages are considered predominantly pro-repair. To reconcile these observations, we propose several alternative interpretations and integrate our results with emerging concepts in macrophage biology and complement-mediated inflammation.

First, M2-like macrophages in this disease context may not function exclusively as reparative cells. Multiple studies have shown that alternatively activated macrophages can contribute to pathological remodeling through fibrogenesis, extracellular matrix deposition, and the production of profibrotic cytokines such as IL-10 and TGF-β. In models of viral pneumonia, ARDS, and ILD, M2-like macrophage expansion has been linked to disease progression rather than resolution (15, 31). These findings suggest that the increased presence of CD206-positive macrophages in anti-MDA5 mAb-associated hepatic injury may reflect, at least in part, macrophage responses that accompany or potentially amplify pathological tissue remodeling.

Second, complement activation likely operates upstream of macrophage recruitment and polarization (32). Complement component C3 deficiency may attenuate both direct complement-mediated cytotoxicity and C3-dependent macrophage chemotaxis or alternative activation. The reduced recruitment of M2-like macrophages observed in C3-deficient mice may therefore indicate suppression of a complement-driven inflammatory amplification loop, rather than a loss of a purely reparative population. This interpretation aligns with evidence that complement fragments such as C3a and C5a can promote macrophage polarization toward profibrotic phenotypes.

Macrophage activation syndrome has been reported as a complication of anti–MDA5 antibody–positive DM, further supporting the concept of macrophage-dominant inflammation in this disease (33). Although C3 deficiency attenuated hepatic injury and reduced M2-like macrophage recruitment in our model, these findings do not establish definitive causal roles for either complement activation or M2-like macrophages. We did not perform interventional studies using complement inhibitors in wild-type mice, nor did we conduct macrophage depletion or adoptive transfer experiments to directly assess the pathogenic potential of M2-like macrophages. Future studies incorporating pharmacologic complement blockade, conditional complement gene deletion, macrophage depletion, or adoptive transfer approaches will be required to more conclusively define the causal contributions of these pathways. Accordingly, complement inhibition or modulation of macrophage polarization may warrant further investigation as adjunctive therapeutic strategies to mitigate pulmonary and hepatic complications in anti–MDA5 antibody–positive DM. However, additional evidence will be required before complement inhibitors can be considered as therapeutic options in this disease.

### Limitations

This study has several important limitations. First, only five autopsy liver samples were available for analysis, representing an extremely small sample size. Because autopsy specimens reflect end-stage disease, the findings may not fully capture pathological changes occurring during earlier or active phases, introducing a potential survival bias.

Second, comparison with normal liver tissue was not feasible because the use of healthy liver samples was not permitted by our institutional ethics committee for ethical reasons. As a result, statements regarding macrophage accumulation are based on observations within diseased tissues and do not involve direct comparison with healthy controls.

Third, CD80-positive and CD206-positive macrophage infiltration was assessed exclusively in autopsy specimens. Because these samples represent end-stage disease, it is not possible to determine whether CD80-positive and CD206-positive macrophages are causative mediators, reactive responders to hepatic injury, or nonspecific features of terminal disease. In addition, differences between our observations and prior biopsy reports cannot be attributed to treatment effects or disease stage with certainty, as no early-stage or longitudinal liver samples were available. These interpretations therefore remain speculative.

Fourth, the single-cell RNA-sequencing analysis was performed using a pooled design, in which liver tissues from three mice per group were combined into a single library. While pooling increases biological representativeness and facilitates the detection of rare cell populations, it precludes the use of formal statistical testing, false discovery rate–based multiple-testing correction, and inferential comparisons of cell-type frequencies or differentially expressed genes between groups. Accordingly, the single-cell RNA-sequencing findings are presented as descriptive and hypothesis-generating rather than inferential.

Fifth, macrophage phenotypes were classified operationally based on marker expression. In the immunohistochemical analysis, M1-like and M2-like macrophages were defined using CD80 and CD206 expression, respectively; however, these markers are not mutually exclusive, and macrophage activation states likely exist along a continuum, particularly in disease contexts. Therefore, the classification of M1-like and M2-like macrophages should be interpreted as a marker-based approximation rather than as discrete, functionally distinct populations.

Sixth, histological assessment of macrophage accumulation was not subjected to formal morphometric quantification. Distinguishing Kupffer cells from infiltrating monocyte-derived macrophages based solely on F4/80 expression is challenging, and overlapping marker expression further limits precise quantification. To address this limitation, quantitative comparisons were instead supported by independent biochemical analyses using western blotting, which provided statistical validation of complement-related and macrophage-associated signals.

Seventh, the control antibodies used in this study consisted of a mixture of mouse IgG subclasses purified from pooled normal serum, whereas the anti-human MDA5 mAbs belonged to the IgG1 or IgG2b subclasses. Differences in IgG subclass composition may alter Fc receptor engagement or complement-activating potential, which could influence the magnitude of antibody effector functions. Although the major comparisons in this study rely on the presence or absence of complement activation (wild-type vs. C3-deficient mice), the subclass heterogeneity of control antibodies should be considered when interpreting the results.

Finally, the experimental model employed in this study is based on acute exogenous administration of anti-human MDA5 mAbs, which does not fully recapitulate the chronic and endogenous autoantibody production observed in patients with anti–MDA5 antibody–positive DM. Accordingly, the present findings should be interpreted as mechanistic insights into antibody–complement–macrophage interactions rather than as a direct phenocopy of the full clinical spectrum of the disease. Future studies incorporating longitudinal sampling, endogenous autoantibody models, or targeted manipulation of macrophage subsets will be required to establish causal relationships and more accurately model disease progression.

## Data Availability

The data presented in this study are deposited in the NCBI Sequence Read Archive (SRA) under BioProject accession number PRJNA1406470. Two independent single-cell RNA sequencing experiments were performed, and all generated data are available in this repository. The samples used for the analyses and figures (**Figure 8**) and **Table 2** presented in this manuscript were included. In addition, an independent replicate dataset using C3-deficient mice, which is not presented in this manuscript, is provided for transparency and reproducibility.

## Supplementary material

Supplementary Figure 1 Anti–human MDA5 polyclonal antibody treatment induced lung and hepatic injury in human MDA5 transgenic mice. Human MDA5 transgenic mice were untreated **(a, b)**, treated with 0.5 mL of control rabbit serum **(c, d)**, or treated with 0.5 mL of anti–human MDA5 polyclonal antibody **(e, f)** on days 0, 7, 14, and 21, and sacrificed on day 28. Lung and liver tissues were analyzed by H&E staining.

Supplementary Figure 2 Anti–human MDA5 polyclonal antibody induced hepatic injury in multiple lines of human MDA5 transgenic mice. Panels **(a, b)** show line 32, **(c, d)** line 55, and **(e, f)** line 116. Mice were treated with 0.5 mL of control rabbit serum **(a, c, e)** or 0.5 mL of anti–human MDA5 polyclonal antibody **(b, d, f)** on days 0, 7, 14, and 21, and sacrificed on day 28. Liver tissues were analyzed by H&E staining.

Supplementary Figure 3 Anti–human MDA5 monoclonal antibody clones induced varying degrees of hepatic injury in human MDA5 transgenic mice (line 55). Line 55 human MDA5 transgenic mice were treated with 0.5 mg of control mouse IgG **(a)** or one of five anti–human MDA5 monoclinal antibody clones—H5 **(b)**, H27 **(c)**, H46 **(d)**, H77 **(e)**, or H85 **(f)**—on days 0, 7, 14, and 21, and sacrificed on day 28. Liver tissues were analyzed by H&E staining.

Supplementary Figure 4 Minimal lung injury following anti–human MDA5 monoclonal antibody treatment in human MDA5 transgenic mice. Mice were treated with 0.5 mg of control mouse IgG **(a)** or the anti–human MDA5 monoclinal antibody mixture **(b)** on days 0, 7, 14, and 21, and sacrificed on day 28. Lung tissues were analyzed by H&E staining.

Supplementary Figure 5 No significant skin injury following treatment with anti–human MDA5 monoclonal or polyclonal antibodies, or control mouse IgG, in human MDA5 transgenic mice. Human MDA5 transgenic mice were administered 0.5 mg of control mouse IgG **(a)**, an anti–human MDA5 monoclinal antibody mixture **(b)**, or 0.5 mL of anti–human MDA5 polyclonal antibody **(c)** on days 0, 7, 14, and 21, and were sacrificed on day 28. Ear skin tissues were analyzed by hematoxylin and eosin (H&E) staining.

Supplementary Figure 6 Serum liver function tests in human MDA5 transgenic and C3-deficient MDA5 transgenic mice. Mice were treated with 0.5 mg of control mouse IgG or 0.5 mg of the anti–human MDA5 monoclinal antibody mixture on days 0, 7, 14, and 21, and sacrificed on day 28. Serum was collected at day 28. Each group included 4–5 mice.

Supplementary Figure 7 Western blot analysis of CD80 and CD206 in liver tissues from human MDA5 transgenic mice with or without C3 deficiency. Representative western blot images of liver tissues from human MDA5 transgenic mice and C3-deficient human MDA5 transgenic mice treated with either anti-human MDA5 monoclinal antibody mixture or control mouse IgG are shown. CD80 and CD206 expression was analyzed on the same blot, with β-actin used as a loading control. Each panel shows independent biological replicates.

## References

[1] Brisse M Ly H . Comparative structure and function analysis of the RIG-I-like receptors: RIG-I and MDA5. Front Immunol. (2019) 10:1586. doi: 10.3389/fimmu.2019.01586, PMID: 31379819 PMC6652118

[2] Dias Junior AG Sampaio NG Rehwinkel J . A balancing act: MDA5 in antiviral immunity and autoinflammation. Trends Microbiol. (2019) 27:75–85. doi: 10.1016/j.tim.2018.08.007, PMID: 30201512 PMC6319154

[3] Oda H Nakagawa K Abe J Awaya T Funabiki M Hijikata A . Aicardi-Goutieres syndrome is caused by IFIH1 mutations. Am J Hum Genet. (2014) 95:121–5. doi: 10.1016/j.ajhg.2014.06.007, PMID: 24995871 PMC4085581

[4] Van Eyck L De Somer L Pombal D Bornschein S Frans G Humblet-Baron S . Brief report: IFIH1 mutation causes systemic lupus erythematosus with selective IgA deficiency. Arthritis Rheumatol. (2015) 67:1592–7. doi: 10.1002/art.39110, PMID: 25777993

[5] Smyth DJ Cooper JD Bailey R Field S Burren O Smink LJ . A genome-wide association study of nonsynonymous SNPs identifies a type 1 diabetes locus in the interferon-induced helicase (IFIH1) region. Nat Genet. (2006) 38:617–9. doi: 10.1038/ng1800, PMID: 16699517

[6] Marie I Hatron PY Dominique S Cherin P Mouthon L Menard JF . Short-term and long-term outcomes of interstitial lung disease in polymyositis and dermatomyositis: a series of 107 patients. Arthritis Rheum. (2011) 63:3439–47. doi: 10.1002/art.30513, PMID: 21702020

[7] Sato S Hirakata M Kuwana M Suwa A Inada S Mimori T . Autoantibodies to a 140-kd polypeptide, CADM-140, in Japanese patients with clinically amyopathic dermatomyositis. Arthritis Rheum. (2005) 52:1571–6. doi: 10.1002/art.21023, PMID: 15880816

[8] Sato S Kuwana M Fujita T Suzuki Y . Amyopathic dermatomyositis developing rapidly progressive interstitial lung disease with elevation of anti-CADM-140/MDA5 autoantibodies. Mod Rheumatol. (2012) 22:625–9. doi: 10.1007/s10165-011-0558-9, PMID: 22124544

[9] Sato S Kuwana M Fujita T Suzuki Y . Anti-CADM-140/MDA5 autoantibody titer correlates with disease activity and predicts disease outcome in patients with dermatomyositis and rapidly progressive interstitial lung disease. Mod Rheumatol. (2013) 23:496–502. doi: 10.1007/s10165-012-0663-4, PMID: 22644102

[10] Yoshida N Kaieda S Tomozoe K Tajiri M Wakasugi D Okamoto M . An autopsy case of anti-melanoma differentiation-associated gene-5 antibody-positive clinical amyopathic dermatomyositis complicated by rapidly progressive interstitial lung disease. Intern Med. (2016) 55:1653–9. doi: 10.2169/internalmedicine.55.6055, PMID: 27301523

[11] Sakamoto S Okamoto M Kaieda S Fujimoto K Nagata S Tominaga M . Low positive titer of anti-melanoma differentiation-associated gene 5 antibody is not associated with a poor long-term outcome of interstitial lung disease in patients with dermatomyositis. Respir Investig. (2018) 56:464–72. doi: 10.1016/j.resinv.2018.07.007, PMID: 30150008

[12] Tsuji H Nakashima R Hosono Y Imura Y Yagita M Yoshifuji H . Multicenter prospective study of the efficacy and safety of combined immunosuppressive therapy with high-dose glucocorticoid, tacrolimus, and cyclophosphamide in interstitial lung diseases accompanied by anti-melanoma differentiation-associated gene 5-positive dermatomyositis. Arthritis Rheumatol. (2020) 72:488–98. doi: 10.1002/art.41105, PMID: 31524333

[13] Nagashima T Kamata Y Iwamoto M Okazaki H Fukushima N Minota S . Liver dysfunction in anti-melanoma differentiation-associated gene 5 antibody-positive patients with dermatomyositis. Rheumatol Int. (2019) 39:901–9. doi: 10.1007/s00296-019-04255-2, PMID: 30790016

[14] Zaizen Y Okamoto M Azuma K Fukuoka J Hozumi H Sakamoto N . Enhanced immune complex formation in the lungs of patients with dermatomyositis. Respir Res. (2023) 24:86. doi: 10.1186/s12931-023-02362-0, PMID: 36934274 PMC10024827

[15] Kaku Y Imaoka H Morimatsu Y Komohara Y Ohnishi K Oda H . Overexpression of CD163, CD204 and CD206 on alveolar macrophages in the lungs of patients with severe chronic obstructive pulmonary disease. PloS One. (2014) 9:e87400. doi: 10.1371/journal.pone.0087400, PMID: 24498098 PMC3907529

[16] Mitsuhashi A Koyama K Ogino H Afroj T Nguyen NT Yoneda H . Identification of fibrocyte cluster in tumors reveals the role in antitumor immunity by PD-L1 blockade. Cell Rep. (2023) 42:112162. doi: 10.1016/j.celrep.2023.112162, PMID: 36870329

[17] Horiike Y Suzuki Y Fujisawa T Yasui H Karayama M Hozumi H . Successful classification of macrophage-mannose receptor CD206 in severity of anti-MDA5 antibody positive dermatomyositis associated ILD. Rheumatol (Oxford). (2019) 58:2143–52. doi: 10.1093/rheumatology/kez185, PMID: 31143953

[18] Okamoto M Zaizen Y Kaieda S Nouno T Koga T Matama G . Soluble form of the MDA5 protein in human sera. Heliyon. (2024) 10:e31727. doi: 10.1016/j.heliyon.2024.e31727, PMID: 38845920 PMC11153190

[19] Hoshino T Kato S Oka N Imaoka H Kinoshita T Takei S . Pulmonary inflammation and emphysema: role of the cytokines IL-18 and IL-13. Am J Respir Crit Care Med. (2007) 176:49–62. doi: 10.1164/rccm.200603-316OC, PMID: 17400729

[20] Kitasato Y Hoshino T Okamoto M Kato S Koda Y Nagata N . Enhanced expression of interleukin-18 and its receptor in idiopathic pulmonary fibrosis. Am J Respir Cell Mol Biol. (2004) 31:619–25. doi: 10.1165/rcmb.2003-0306OC, PMID: 15308504

[21] Takenaka SI Kaieda S Kawayama T Matsuoka M Kaku Y Kinoshita T . IL-38: A new factor in rheumatoid arthritis. Biochem Biophys Rep. (2015) 4:386–91. doi: 10.1016/j.bbrep.2015.10.015, PMID: 29124228 PMC5669445

[22] Su Q Kim SY Adewale F Zhou Y Aldler C Ni M . Single-cell RNA transcriptome landscape of hepatocytes and non-parenchymal cells in healthy and NAFLD mouse liver. iScience. (2021) 24:103233. doi: 10.1016/j.isci.2021.103233, PMID: 34755088 PMC8560975

[23] Cao L Wu D Qin L Tan D Fan Q Jia X . Single-cell RNA transcriptome profiling of liver cells of short-term alcoholic liver injury in mice. Int J Mol Sci. (2023) 24(5):4344. doi: 10.3390/ijms24054344, PMID: 36901774 PMC10002329

[24] Nombel A Fabien N Coutant F . Dermatomyositis with anti-MDA5 antibodies: bioclinical features, pathogenesis and emerging therapies. Front Immunol. (2021) 12:773352. doi: 10.3389/fimmu.2021.773352, PMID: 34745149 PMC8564476

[25] Kissel JT Mendell JR Rammohan KW . Microvascular deposition of complement membrane attack complex in dermatomyositis. N Engl J Med. (1986) 314:329–34. doi: 10.1056/NEJM198602063140601, PMID: 3945256

[26] Mascaro JM Jr. Hausmann G Herrero C Grau JM Cid MC Palou J . Membrane attack complex deposits in cutaneous lesions of dermatomyositis. Arch Dermatol. (1995) 131:1386–92. doi: 10.1001/archderm.1995.01690240040007, PMID: 7492126

[27] Pestronk A Schmidt RE Choksi R . Vascular pathology in dermatomyositis and anatomic relations to myopathology. Muscle Nerve. (2010) 42:53–61. doi: 10.1002/mus.21651, PMID: 20544925

[28] Lahoria R Selcen D Engel AG . Microvascular alterations and the role of complement in dermatomyositis. Brain. (2016) 139:1891–903. doi: 10.1093/brain/aww122, PMID: 27190020

[29] Beljaars L Schippers M Reker-Smit C Martinez FO Helming L Poelstra K . Hepatic localization of macrophage phenotypes during fibrogenesis and resolution of fibrosis in mice and humans. Front Immunol. (2014) 5:430. doi: 10.3389/fimmu.2014.00430, PMID: 25250030 PMC4157549

[30] Krenkel O Tacke F . Liver macrophages in tissue homeostasis and disease. Nat Rev Immunol. (2017) 17:306–21. doi: 10.1038/nri.2017.11, PMID: 28317925

[31] Gieseck RL 3rd Wilson MS Wynn TA . Type 2 immunity in tissue repair and fibrosis. Nat Rev Immunol. (2018) 18:62–76. doi: 10.1038/nri.2017.90, PMID: 28853443

[32] Ricklin D Lambris JD . Complement in immune and inflammatory disorders: pathophysiological mechanisms. J Immunol. (2013) 190:3831–8. doi: 10.4049/jimmunol.1203487, PMID: 23564577 PMC3623009

[33] Ding Y Ge Y . Anti-melanoma differentiation-associated gene 5 antibody-positive dermatomyositis complicated with macrophage activation syndrome. Ther Adv Chronic Dis. (2022) 13:20406223221098128. doi: 10.1177/20406223221098128, PMID: 35586303 PMC9109495

