# Supplementary figures and images for: Complement activation and M2-like macrophage accumulation in anti-MDA5 monoclonal antibody–induced hepatic injury in mice
Source: Front Immunol. 2026 Feb 2;17:1707202. doi: 10.3389/fimmu.2026.1707202 (PMC12907824; doi:10.3389/fimmu.2026.1707202)

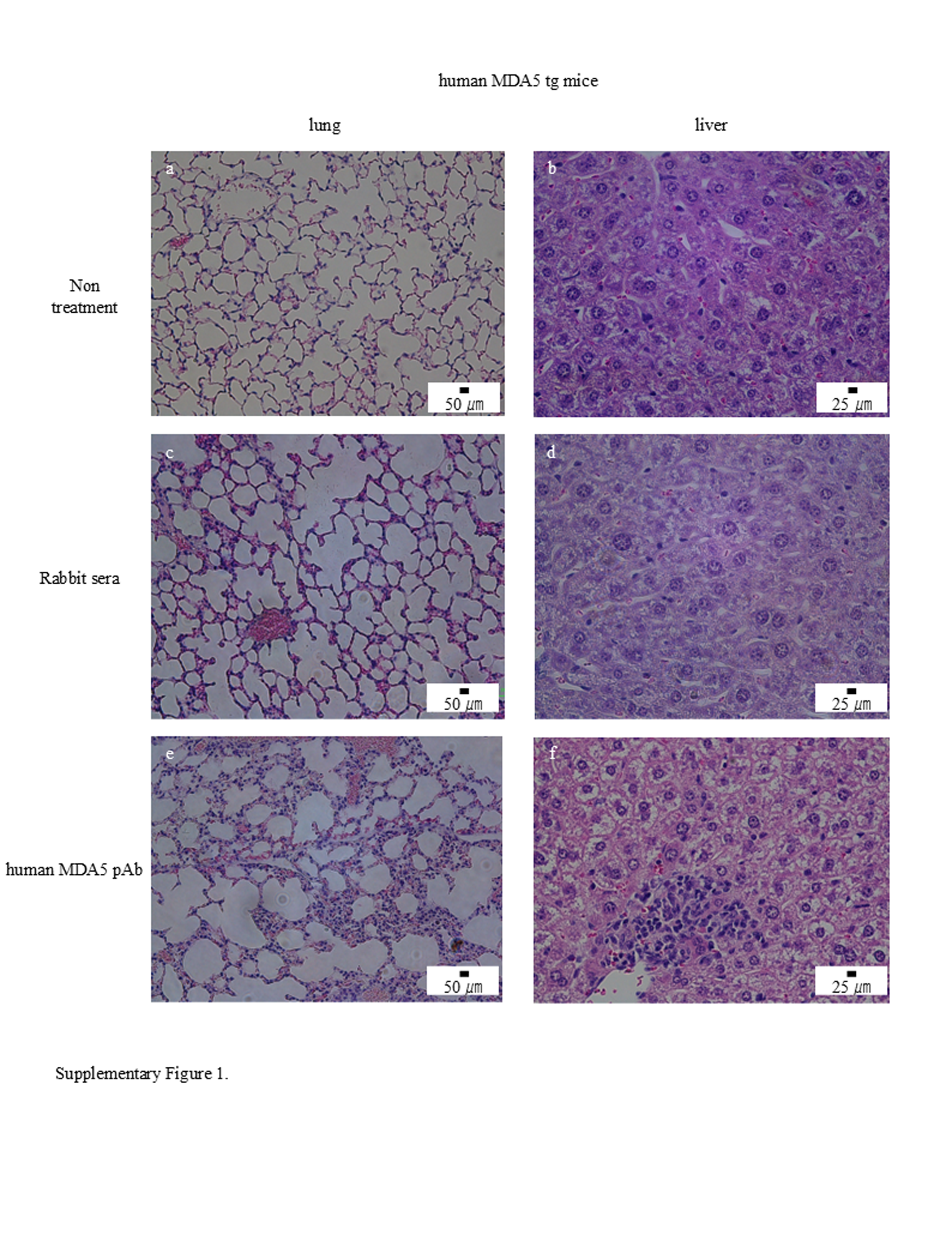

Supplement: Supplementary Figure 1 — Anti–human MDA5 polyclonal antibody treatment induced lung and hepatic injury in human MDA5 transgenic mice. Human MDA5 transgenic mice were untreated (a, b), treated with 0.5 mL of control rabbit serum (c, d), or treated with 0.5 mL of anti–human MDA5 polyclonal antibody (e, f) on days 0, 7, 14, and 21, and sacrificed on day 28. Lung and liver tissues were analyzed by H&E staining. [file Image1.tif]

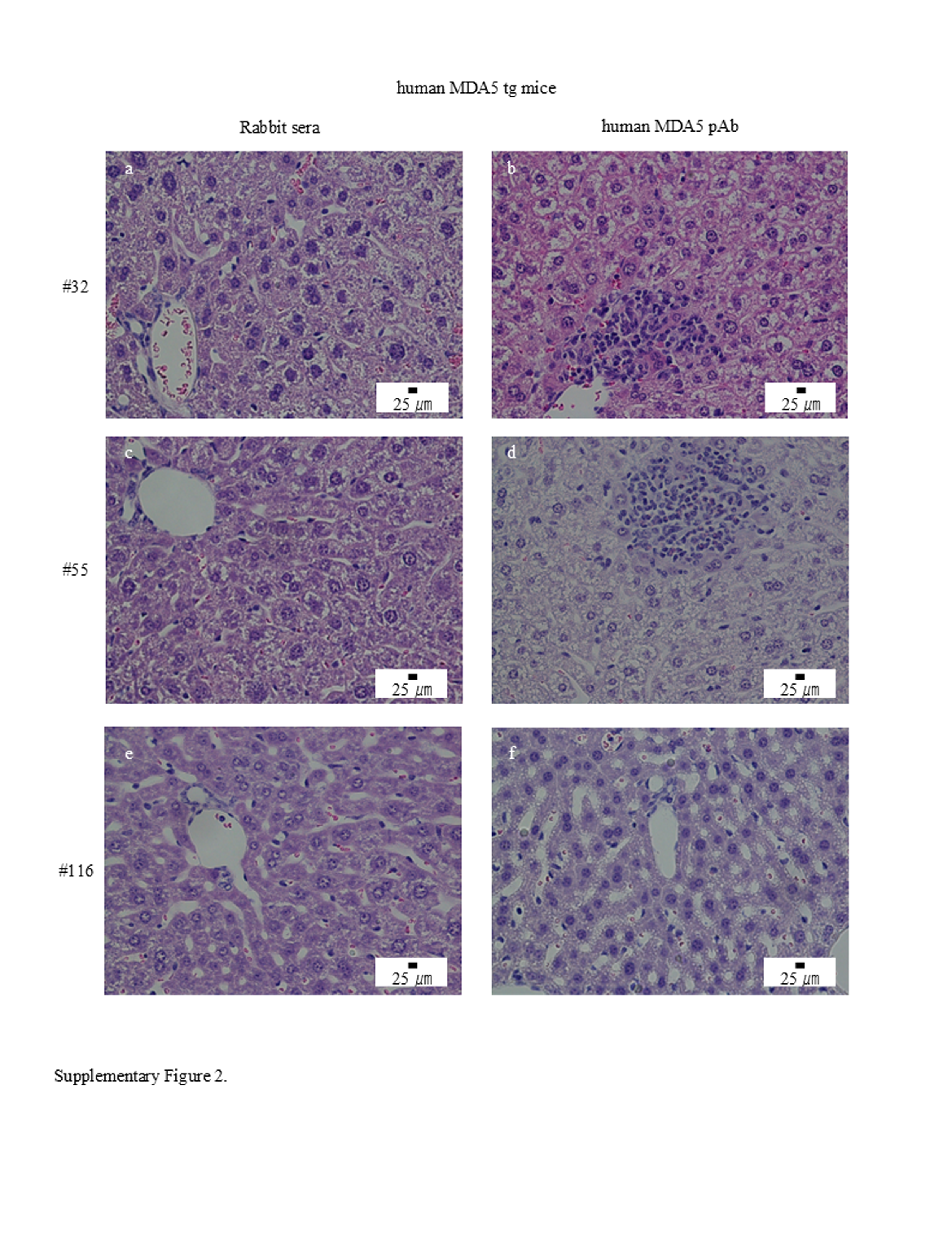

Supplement: Supplementary Figure 2 — Anti–human MDA5 polyclonal antibody induced hepatic injury in multiple lines of human MDA5 transgenic mice. Panels (a, b) show line 32, (c, d) line 55, and (e, f) line 116. Mice were treated with 0.5 mL of control rabbit serum (a, c, e) or 0.5 mL of anti–human MDA5 polyclonal antibody (b, d, f) on days 0, 7, 14, and 21, and sacrificed on day 28. Liver tissues were analyzed by H&E staining. [file Image2.tif]

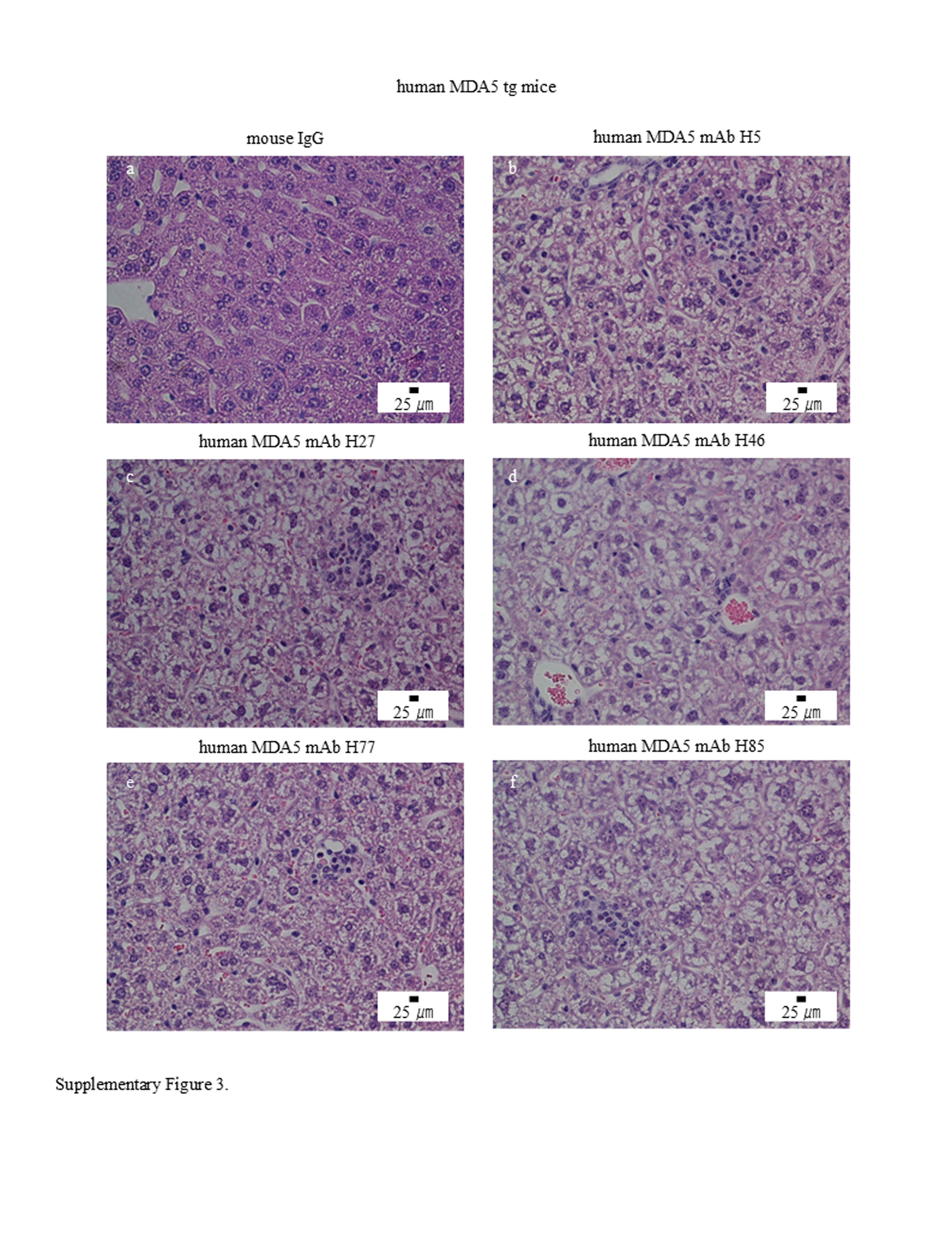

Supplement: Supplementary Figure 3 — Anti–human MDA5 monoclonal antibody clones induced varying degrees of hepatic injury in human MDA5 transgenic mice (line 55). Line 55 human MDA5 transgenic mice were treated with 0.5 mg of control mouse IgG (a) or one of five anti–human MDA5 monoclinal antibody clones—H5 (b), H27 (c), H46 (d), H77 (e), or H85 (f)—on days 0, 7, 14, and 21, and sacrificed on day 28. Liver tissues were analyzed by H&E staining. [file Image3.tif]

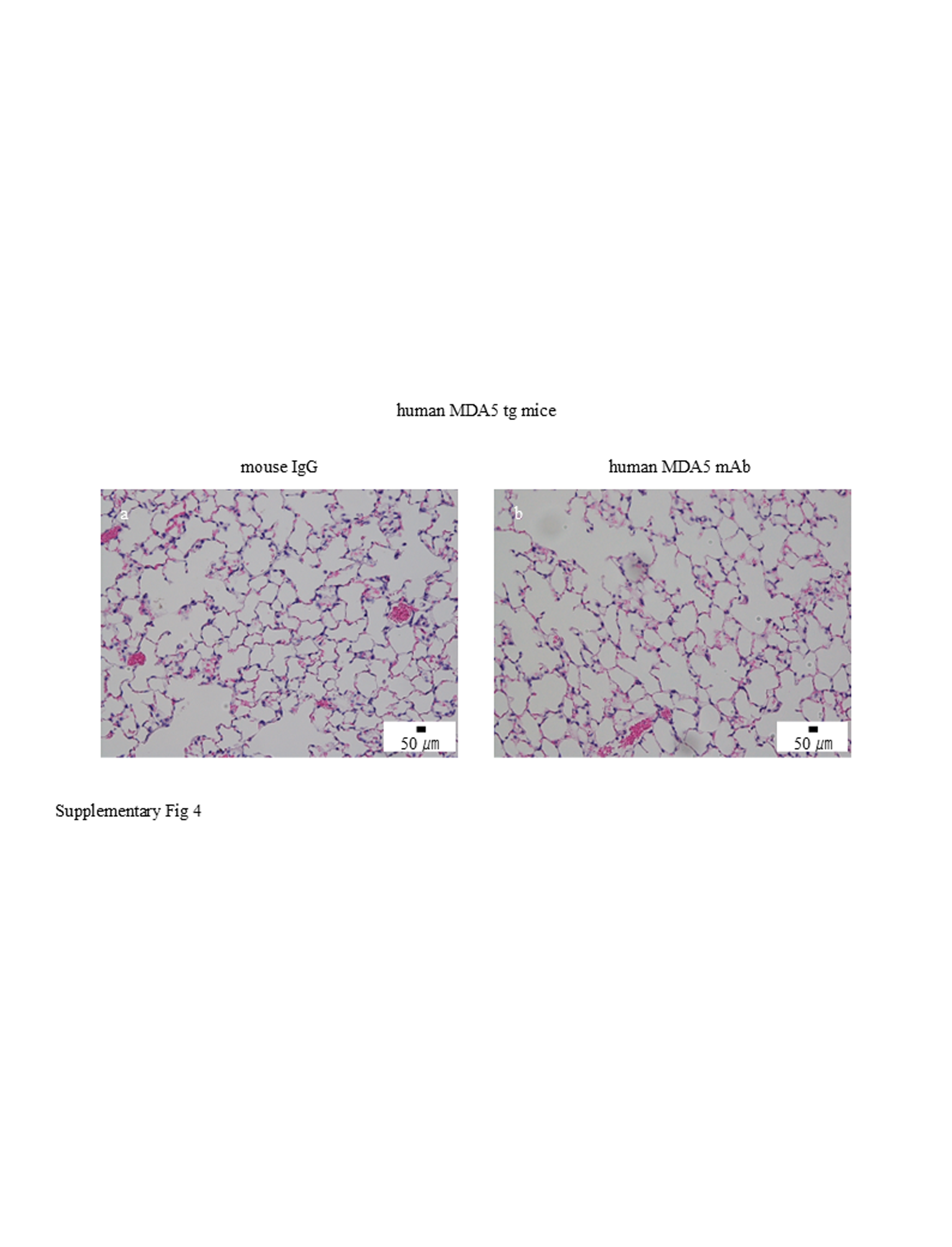

Supplement: Supplementary Figure 4 — Minimal lung injury following anti–human MDA5 monoclonal antibody treatment in human MDA5 transgenic mice. Mice were treated with 0.5 mg of control mouse IgG (a) or the anti–human MDA5 monoclinal antibody mixture (b) on days 0, 7, 14, and 21, and sacrificed on day 28. Lung tissues were analyzed by H&E staining. [file Image4.tif]

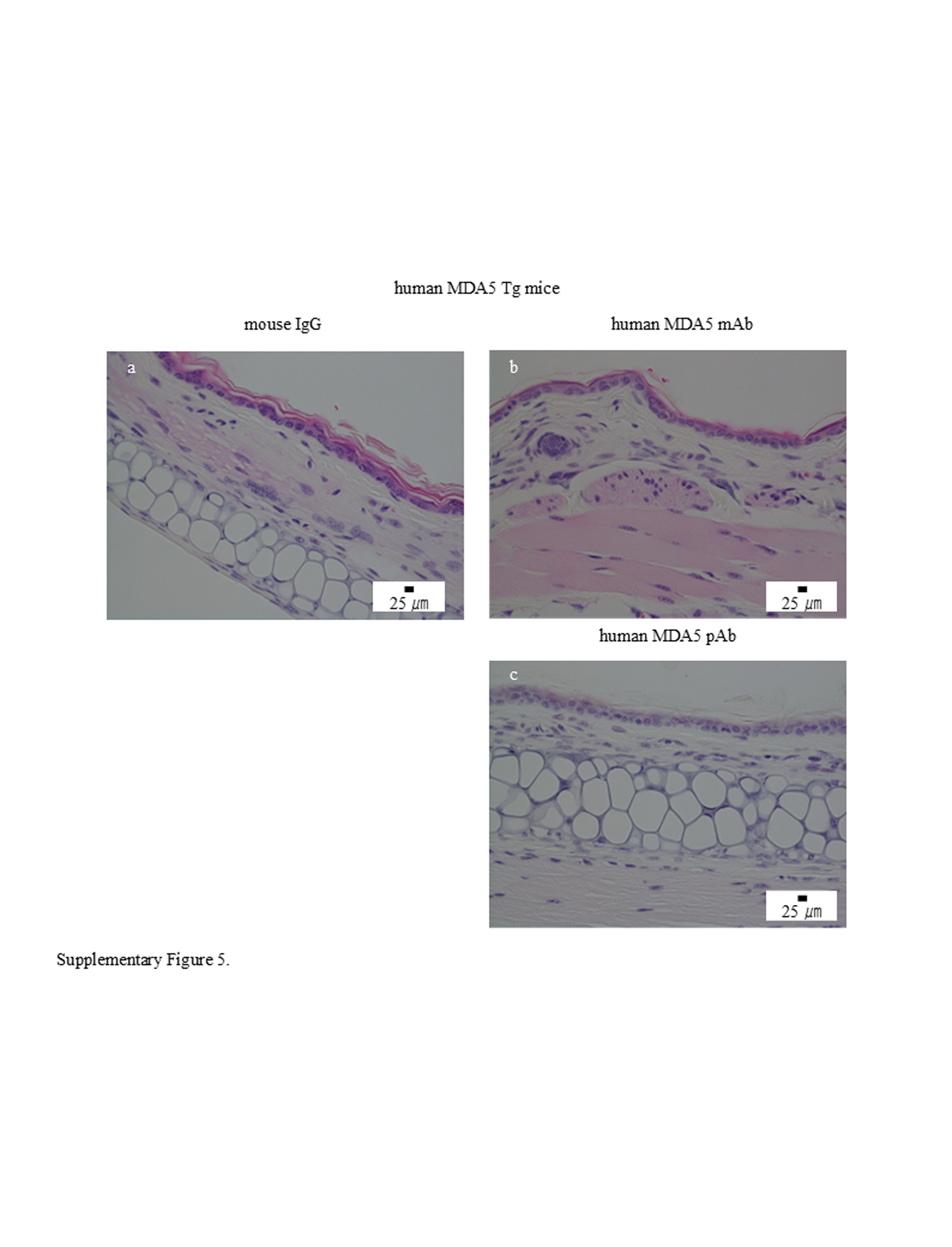

Supplement: Supplementary Figure 5 — No significant skin injury following treatment with anti–human MDA5 monoclonal or polyclonal antibodies, or control mouse IgG, in human MDA5 transgenic mice. Human MDA5 transgenic mice were administered 0.5 mg of control mouse IgG (a), an anti–human MDA5 monoclinal antibody mixture (b), or 0.5 mL of anti–human MDA5 polyclonal antibody (c) on days 0, 7, 14, and 21, and were sacrificed on day 28. Ear skin tissues were analyzed by hematoxylin and eosin (H&E) staining. [file Image5.tif]

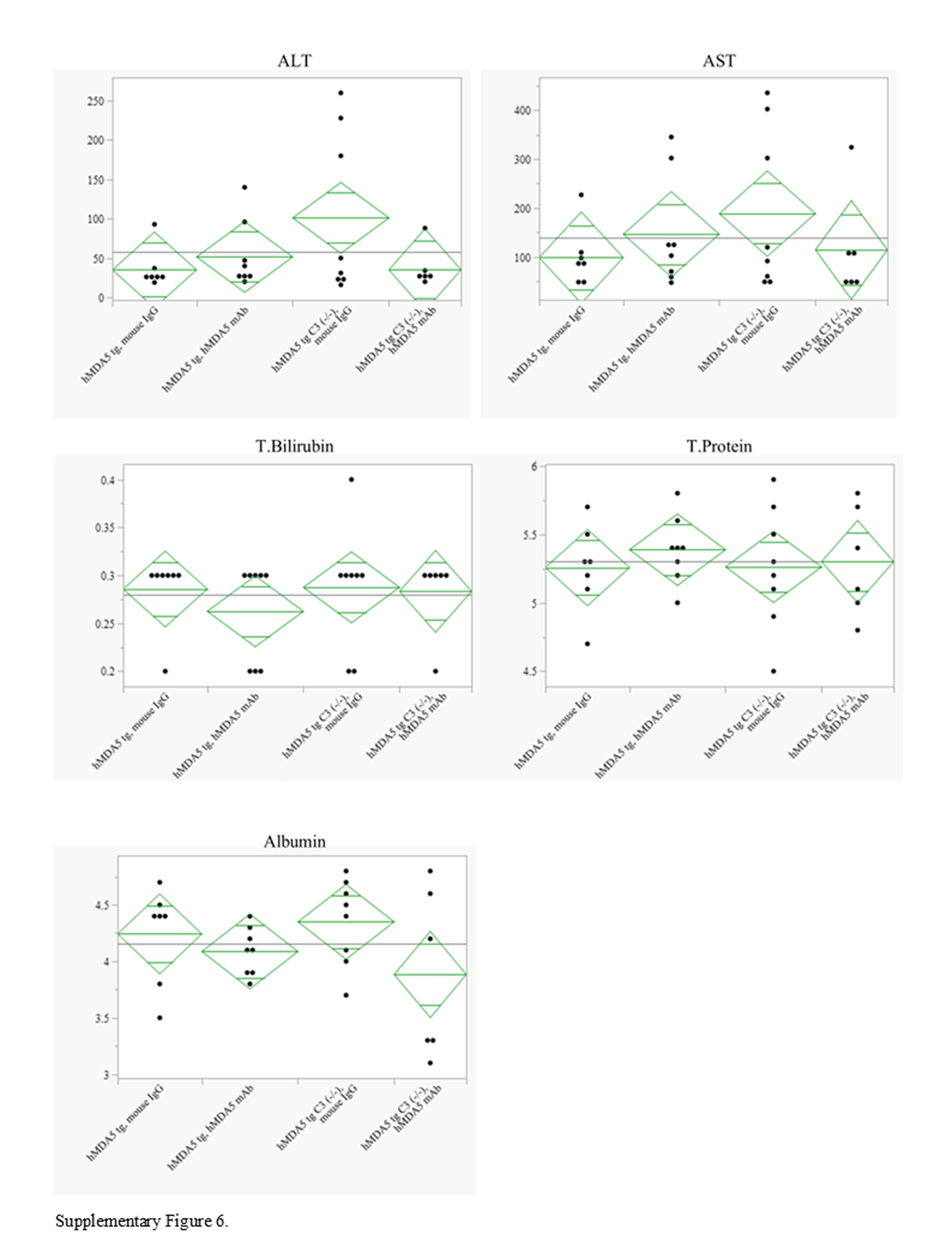

Supplement: Supplementary Figure 6 — Serum liver function tests in human MDA5 transgenic and C3-deficient MDA5 transgenic mice. Mice were treated with 0.5 mg of control mouse IgG or 0.5 mg of the anti–human MDA5 monoclinal antibody mixture on days 0, 7, 14, and 21, and sacrificed on day 28. Serum was collected at day 28. Each group included 4–5 mice. [file Image6.tif]

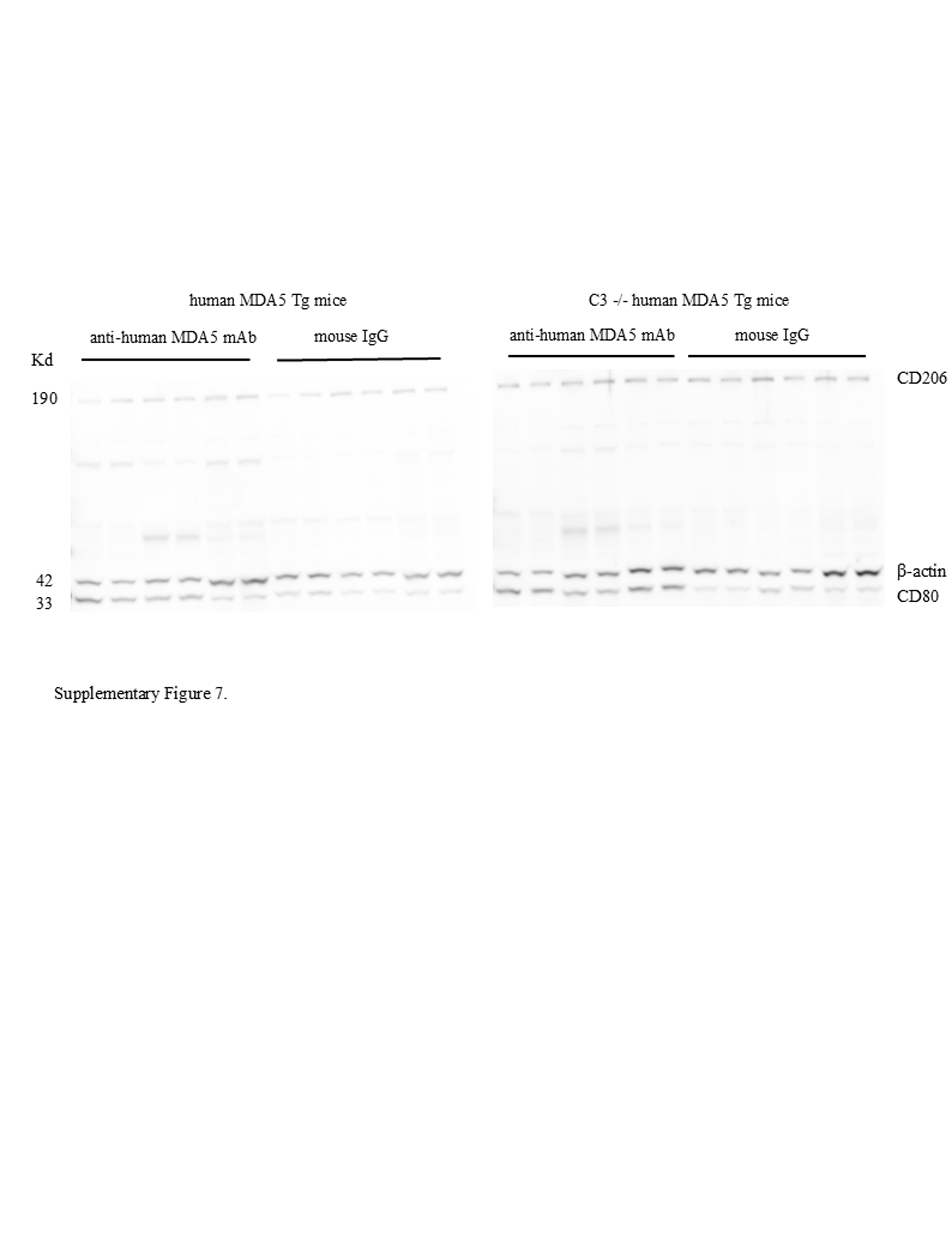

Supplement: Supplementary Figure 7 — Western blot analysis of CD80 and CD206 in liver tissues from human MDA5 transgenic mice with or without C3 deficiency. Representative western blot images of liver tissues from human MDA5 transgenic mice and C3-deficient human MDA5 transgenic mice treated with either anti-human MDA5 monoclinal antibody mixture or control mouse IgG are shown. CD80 and CD206 expression was analyzed on the same blot, with β-actin used as a loading control. Each panel shows independent biological replicates. [file Image7.tif]
